# Supplementary material for: gNOMO2: a comprehensive and modular pipeline for integrated multi-omics analyses of microbiomes
Source: Gigascience. 2024 Jul 12;13:giae038. doi: 10.1093/gigascience/giae038 (PMC11240238; doi:10.1093/gigascience/giae038)
Supplement: giae038_GIGA-D-24-00028_Revision_1 [file giae038_giga-d-24-00028_revision_1.pdf]

GigaScience

gNOMO2: a comprehensive and modular pipeline for integrated multi-omics analyses of microbiomes

--Manuscript Draft--

**Manuscript Number:**	GIGA-D-24-00028R1
**Full Title:**	gNOMO2: a comprehensive and modular pipeline for integrated multi-omics analyses of microbiomes
**Article Type:**	Research
**Funding Information:**	Türkiye Bilimsel ve Teknolojik Araştırma Kurumu — Dr. Muzaffer Arıkan
**Abstract:**	Background<br><br>In recent years, omics technologies has offered an exceptional chance to gain a deeper insight into the structural and functional characteristics of microbial communities. As a result, there is a growing demand for user friendly, reproducible, and versatile bioinformatic tools that can effectively harness multi-omics data to offer a holistic understanding of microbiomes. Previously, we introduced gNOMO, a bioinformatic pipeline tailored to analyze microbiome multi-omics data in an integrative manner. In response to the evolving demands within the microbiome field and the growing necessity for integrated multi-omics data analysis, we have implemented substantial enhancements to the gNOMO pipeline.<br><br>Results<br><br>Here, we present gNOMO2, a comprehensive and modular pipeline that can seamlessly manage various omics combinations, ranging from two to four distinct omics data types including 16S rRNA gene amplicon sequencing, metagenomics, metatranscriptomics, and metaproteomics. Furthermore, gNOMO2 features a specialized module for processing 16S rRNA gene amplicon sequencing data to create a protein database suitable for metaproteomics investigations. Moreover, it incorporates new differential abundance, integration and visualization approaches, enhancing the toolkit for a more insightful analysis of microbiomes. The functionality of these new features is showcased through the use of four microbiome multi-omics datasets encompassing various ecosystems and omics combinations. gNOMO2 not only replicated most of the primary findings from these studies but also offered further valuable perspectives.<br><br>Conclusions<br><br>gNOMO2 enables the thorough integration of taxonomic and functional analyses in microbiome multi-omics data, offering novel insights in both host associated and free-living microbiome research. gNOMO2 is available freely at https://github.com/muzafferarikan/gNOMO2.
**Corresponding Author:**	Muzaffer Arıkan<br>Istanbul Medipol University: Istanbul Medipol Universitesi<br>Istanbul, TURKEY
**Corresponding Author Secondary Information:**	
**Corresponding Author's Institution:**	Istanbul Medipol University: Istanbul Medipol Universitesi
**Corresponding Author's Secondary Institution:**	
**First Author:**	Muzaffer Arıkan
**First Author Secondary Information:**	
**Order of Authors:**	Muzaffer Arıkan

Powered by Editorial Manager® and ProduXion Manager® from Aries Systems Corporation

	Thilo Muth
**Order of Authors Secondary Information:**	
**Response to Reviewers:**	We would first like to thank the editor and reviewers for their thoughtful and valuable feedback. Please find below our responses to each of comments or questions.<br><br>Reviewer #1:<br>"gNOMO2: a comprehensive and modular pipeline for integrated multi-omics analyses of microbiomes" by Arıkan and Muth presents a multi-omics tools for analysis of prokaryotes. It is an evolution of the first version and offers various separate modules, taking different type of input data. They present different example analysis based on already published data and reproduced the results. The manuscript is very well written (I could not detect a single typo) and it was fun to read! Well done! I have only very few comments and suggestions, see below. However, I had a problem executing the code.<br><br>We sincerely thank the Reviewer for taking time to review our manuscript and for valuable comments. Please find below our responses to each of your comments or questions.<br><br><br>################<br><br>Key questions to answer:<br><br>1) Are the methods appropriate to the aims of the study, are they well described, and are necessary controls included?<br>Yes<br>2) Are the conclusions adequately supported by the data shown?<br>Yes<br>3) Please indicate the quality of language in the manuscript. Does it require a heavy editing for language and clarity?<br>Very well written!<br>4) Are you able to assess all statistics in the manuscript, including the appropriateness of statistical tests used?<br>No direct statistics given in the manuscript. Maybe the authors could include some example output as .zip file for interested potential users.<br>We uploaded an example output file for statistical analysis results to GigaDB data repository.<br><br>################<br><br>Detailed comments to the manuscript:<br><br>Line 168: What does "cleaned and redundancies are removed" mean? Are only identical genomes removed? Or are genome part that are identical (I guess this barely exists, except for conserved gene parts as 16S, or similar) removed? Or are only redundant genes removed? How is redundancy defined, 99% identical stretch?<br>gNOMO2 retains only a single protein sequence from identical protein sequences, effectively removing redundancy among protein sequences that are 100% identical.<br>We revised the sentence as follows:<br>“All downloaded sequences are merged and cleaned, and a single protein sequence is retained from identical protein sequences to effectively remove redundancy using SeqKit [26].”<br><br>Line 399-405: When looking at figure 5A I am wondering how Fluviicoccus and Methanosarcina in the MP faction appear relatively abundant in some samples. Where they de novo assembled in the MG or MT modules?<br>We identified Fluviicoccus and Methanosarcina in both the MG and MT datasets. However, their relative abundances were comparatively low and thus are not displayed in Figure 5A, which showcases only the top 10 most abundant taxa. However, the taxonomic abundance tables based on MG and MT have been uploaded to the GigaDB data repository with file names “herold_taxa_abundance_mg.txt” and “herold_taxa_abundance_mt.txt”, respectively.

Powered by Editorial Manager® and ProduXion Manager® from Aries Systems Corporation

General comment figures: I know that it is a hack to deal with automatic figure generation and especially the axis labels (as names have very different length). However, I think some figures might be hardly visible in the printed version, especially axes label for panel B are very small. Maybe you can put the critical figures separately in the supplement, e.g. each B panel a one page.
We have uploaded each panel of the figures separately as high-quality figures to GigaDB data repository.

Suggestions: As suggest above, maybe the authors could include some example output (a simple example) as .zip file for interested potential users. This would give an idea of how the output looks like and what to expect besides the plots. But differential abundance tables might be more important than the plots, as the user would generate their own plot for later publications.
We have provided plots, metadata tables, phyloseq objects containing sample data, taxonomy tables, and abundance tables for each dataset analyzed in our manuscript as supplementary data files to GigaDB repository.

################

Github & software:

I also tested the software and followed the instructions in the Github. I successfully executed the "Requirements" and "Config" steps (including create of metadata file and copying of amplicon reads) and tried to execute Modul1. However, the following error occurred (using up-to-date conda and snakemake on Ubuntu linux):

(snakemake) abartho@gmbs17:~/review_papers/GigaScience/gNOMO2$ snakemake -v
6.15.5

(snakemake) abartho@gmbs17:~/review_papers/GigaScience/gNOMO2$ snakemake -s workflow/Snakefile --cores 20
SyntaxError in line 9 of /home/abartho/miniconda3/envs/snakemake/lib/python3.6/site-packages/smart_open/s3.py:
future feature annotations is not defined (s3.py, line 9)
File "/home/abartho/miniconda3/envs/snakemake/lib/python3.6/site-packages/smart_open/__init__.py", line 34, in <module>
File "/home/abartho/miniconda3/envs/snakemake/lib/python3.6/site-packages/smart_open/smart_open_lib.py", line 35, in <module>
File "/home/abartho/miniconda3/envs/snakemake/lib/python3.6/site-packages/smart_open/doctools.py", line 21, in <module>
File "/home/abartho/miniconda3/envs/snakemake/lib/python3.6/site-packages/smart_open/transport.py", line 104, in <module>
File "/home/abartho/miniconda3/envs/snakemake/lib/python3.6/site-packages/smart_open/transport.py", line 49, in register_transport
File "/home/abartho/miniconda3/envs/snakemake/lib/python3.6/importlib/__init__.py", line 126, in import_module

In addition to solving the problem, an example metadata file and some explanation about the output (which I did not see yet) would be good for less experienced users.
This error suggests that the Python version you are using (3.6) does not support future annotations, which were introduced in Python 3.7. Snakemake versions up to 6.0.6 are compatible with Python 3.6. Starting from version 6.1.0, Snakemake requires Python 3.7 or later. Your Snakemake version (6.15.5) is incompatible with Python 3.6 installed on your system. To resolve this, you can upgrade your Python version to 3.7 or later. Additionally, we have updated the gNOMO2 code to require a specific version of Snakemake instead of a minimum version to avoid any other potential compatibility problems.

Reviewer #2:
This paper introduced gNOMO2, a new version of gNOMO, which is a bioinformatic pipeline for multiomic management and analysis of microbiomes. The authors claimed that gNOMO2 incorporates new differential abundance, integration, and visualization

tools compared to gNOMO. However, these new features as well as the distinction between gNOMO2 and gNOMO has not been clearly presented in the paper. In addition, the Methods section is written as a pipeline of bioinformatic tools and it is not clear what these tools are used for unless one is familiar with all the bioinformatic tools.
We sincerely thank the Reviewer for taking time to review our manuscript and for providing valuable comments. Please find below our responses to each of your comments or questions.

My major comments are as follows:

1.Given the existing work on gNOMO, it is critical for the authors to distinguish gNOMO2 from gNOMO to show its novelty. In the Methods section, the authors presented the six modules of gNOMO2. Are these all new from gNOMO, or does gNOMO included some of these functions? A clearer presentation of gNOMO2 versus gNOMO is needed.
We have included the following paragraph in the Methods section to elucidate the distinctions between gNOMO and gNOMO2:
“The original gNOMO accepts MG, MT and MP data as input and generates results for differential abundance analysis in each omics layer. It also constructs a protein database using MG and MT data and performs both differential abundance and pathway level integrated analyses (Figure 1A). In contrast, gNOMO2 pipeline comprises six modules that facilitate direct analysis of various omics combinations. Each module includes pre-processing, analysis of each omics dataset, data integration and visualization steps (Figure 1B). We implemented changes to both the analysis workflow and pipeline structure. For workflow adjustments, we updated the quality control, merging, assembly, differential abundance, and visualization steps. In the quality control phase, we switched from using PrinSeq to Trimmomatic for cleaning and trimming reads. For read merging, we replaced fastq-join with FLASH to merge paired-end reads. In the assembly step, we transitioned from Ray to metaSPAdes for de novo assembly of metagenomic sequences, and from Ray to rnaSPAdes for de novo assembly of metatranscriptomic sequences. In the differential abundance analysis step, we replaced LefSe with MaAsLin2. For visualization, we replaced Krona with ggplot2 to analyze taxonomic composition, enabling combined visualization of samples. We also replaced LefSe with MaAsLin2 for visualizing the results of differential abundance analysis. For pathway-level analysis results, we kept Pathview unchanged, but for joint-visualization analysis, we used the combi package to visualize outputs. These workflow changes and comparisons between gNOMO and gNOMO2 are depicted in Supplementary Fig. S1.

Additionally, we introduced changes to facilitate the incorporation of metadata tables into analyses and automated the creation of the configuration file. To enhance and update the structure of the original gNOMO, we implemented six modules in the new gNOMO2 pipeline, allowing for the processing of different omics combinations. The original gNOMO pipeline consisted of only one module (Module 5 in gNOMO2), while gNOMO2 introduced five more modules for specific combinations, along with the ability to accept AS data as input.”

We also added Supplementary Fig. S1 to illustrate the step-by-step differences between the analysis workflow conducted by gNOMO and gNOMO2.

2.The authors did not present the methods in each module very well. For example, the authors wrote in Module 2 that "MaAsLin2 [31] is employed to determine differentially abundant taxa based on both AS and MP data. Furthermore, a joint visualization of MP and AS results is performed using the combi R package [32]. The final outputs include AS and MP based abundance tables, results from differential abundance analysis, and joint visualization analysis results." Without reading the references 31 and 32, it is very hard to understand what this module is really doing.
We have revised the Methods section to provide more detailed explanations of the analyses conducted in each module. The tools, analyses, and outputs are now explained more clearly.

3.The authors used the term "integrated multi-omics analysis" in all six modules of

	gNOMO2. It is not clear how this terms really means. It reads like that it is not really integrated analysis, instead, it is more like a module that can handle different types of data separately, such as differential abundance analysis for each type. What other integration has been used except joint visualization? What new integration tools have been incorporated in gNOMO2?<br>gNOMO2 employs both middle and late integration strategies in its multi-omics microbiome analysis. Pathway and differential abundance analysis-based integrative approaches are considered late integration strategies, where each omics dataset is analyzed separately and then the results are combined. These analyses are utilized in modules 3-6. In contrast, joint-visualization based integration is a middle integration approach that integrates different omics data according to a predefined model, analyzes them together, and generates the outputs. This approach is employed in all modules except Module 1. Additionally, gNOMO2 incorporates integrative analysis of nucleic acid sequencing-based omics methods with metaproteomics data by creating a proteogenomic database, which is utilized in modules 2-6. While the specific integrative analysis methods vary between modules, gNOMO2 ensures that at least one integrative analysis is included in every module.<br><br>4.In the differential abundance analysis, does the pipeline consider the features of microbiome data, such as their count, sparsity, and compositional features? Can the modules incorporate covariates in their differential abundance analysis? It is quite useful to have covariates adjusted in a differential abundance analysis?<br>We have updated gNOMO2 with a new feature in the configuration file that enables users to select the normalization (Choices: "TSS", "CLR", "CSS", "NONE", "TMM") or transformation (Choices: "LOG", "LOGIT", "AST", "NONE") to apply prior to conducting the differential abundance analysis. Additionally, we have introduced a new parameter that allows users to include covariates listed in the metadata table in the differential abundance analysis.<br><br><br>5.In the Analyses section, the authors applied gNOMO2 to re-analyze samples from previously published studies. They found some discrepancy between their results and the ones in the literature. Although some discrepancy is normal, the authors need to explain better what causes the discrepancy and whether it could yield different biological conclusions.<br>We detected some discrepancies between gNOMO2 results and those reported by Duru et al. (2018) and Herold et al. (2020). To address this, we have added the following paragraphs to the Analyses section to explain better the potential reasons and consequences for these discrepancies:<br><br>For Duru et. al (2018):<br>“Our findings emphasize the critical role of accurately interpreting analysis outcomes based on the structure of the analytical pipeline. Assuming a default approach, particularly during comparison steps, could lead to unsupported conclusions. In meta-omics studies, various approaches can be employed for data analysis. While none of these approaches are inherently wrong, they may not align with the goals set by the research group. When the pipeline's structure is well-defined, no inconsistencies in biological conclusions would be expected. Additionally, we stress the importance of clear language in explaining results in research articles, as failure to do so may mislead readers. In this instance, the discrepancy was primarily due to differences between the approach depending on comparisons at individual MAG level and the gNOMO2 approach, which compares with the whole community.”<br><br>For Herold et. al (2020):<br>“Our findings highlight that read-based and MAG-based taxonomic composition analysis approaches can lead to divergent results and interpretations. Since neither approach is inherently wrong, this disparity underscores the significance and advantage of thoroughly examining meta-omics datasets using various methodologies. Hence, we underscore that employing diverse approaches and perspectives in complex multi-omics datasets may reveal novel insights extending beyond the original hypothesis.”
**Additional Information:**	
**Question**	**Response**

Are you submitting this manuscript to a special series or article collection?	Yes
Please select an option from the menu:<br>as follow-up to "Are you submitting this manuscript to a special series or article collection?"	Functional Metagenomics
**Experimental design and statistics**<br><br>Full details of the experimental design and statistical methods used should be given in the Methods section, as detailed in our Minimum Standards Reporting Checklist. Information essential to interpreting the data presented should be made available in the figure legends.<br><br>Have you included all the information requested in your manuscript?	Yes
**Resources**<br><br>A description of all resources used, including antibodies, cell lines, animals and software tools, with enough information to allow them to be uniquely identified, should be included in the Methods section. Authors are strongly encouraged to cite Research Resource Identifiers (RRIDs) for antibodies, model organisms and tools, where possible.<br><br>Have you included the information requested as detailed in our Minimum Standards Reporting Checklist?	Yes
**Availability of data and materials**<br><br>All datasets and code on which the conclusions of the paper rely must be either included in your submission or deposited in publicly available repositories (where available and ethically appropriate), referencing such data using a unique identifier in the references and in the “Availability of Data and Materials”	Yes

section of your manuscript.<br><br>Have you have met the above requirement as detailed in our Minimum Standards Reporting Checklist?	

Powered by Editorial Manager® and ProduXion Manager® from Aries Systems Corporation

Manuscript

Click here to access/download;Manuscript;gnomo2-paper-draft-v22.docx

gNOMO2: a comprehensive and modular pipeline for integrated multi-omics analyses of microbiomes

Muzaffer Arıkan[1,2*] and Thilo Muth[3*]

[1] Regenerative and Restorative Medicine Research Center (REMER), Research Institute for Health Sciences and Technologies (SABITA), Istanbul Medipol University, Istanbul, Türkiye

[2] Department of Medical Biology, Faculty of Medicine, Istanbul Medipol University, Istanbul, Türkiye

[3] Domain Data Competence Center (MF 2), Robert Koch Institute (RKI), Berlin, Germany

[*] Correspondence: muzafferarikan@gmail.com and mutht@rki.de

Abstract

Background: In recent years, omics technologies has offered an exceptional chance to gain a deeper insight into the structural and functional characteristics of microbial communities. As a result, there is a growing demand for user friendly, reproducible, and versatile bioinformatic tools that can effectively harness multi-omics data to offer a holistic understanding of microbiomes. Previously, we introduced gNOMO, a bioinformatic pipeline tailored to analyze microbiome multi-omics data in an integrative manner. In response to the evolving demands within the microbiome field and the growing necessity for integrated multi-omics data analysis, we have implemented substantial enhancements to the gNOMO pipeline.

Results: Here, we present gNOMO2, a comprehensive and modular pipeline that can seamlessly manage various omics combinations, ranging from two to four distinct omics data types including 16S rRNA gene amplicon sequencing, metagenomics, metatranscriptomics, and metaproteomics. Furthermore, gNOMO2 features a specialized module for processing 16S rRNA gene amplicon sequencing data to create a protein database suitable for metaproteomics investigations. Moreover, it incorporates new differential abundance, integration and visualization approaches, enhancing the toolkit for a more insightful analysis of microbiomes. The functionality of these new features is showcased through the use of four microbiome multi-omics datasets encompassing various ecosystems and omics combinations. gNOMO2 not only replicated most of the primary findings from these studies but also offered further valuable perspectives.

Conclusions: gNOMO2 enables the thorough integration of taxonomic and functional analyses in microbiome multi-omics data, offering novel insights in both host associated and free-living microbiome research. gNOMO2 is available freely at https://github.com/muzafferarikan/gNOMO2.

Keywords: microbiome, multi-omics, data integration, amplicon sequencing, metagenomics, metatranscriptomics, metaproteomics

Background

Microbiomes play pivotal roles in shaping the environments they inhabit such as influencing host health and disease [1], and contributing to the overall diversity of life on Earth [2]. The comprehensive understanding of microbial communities and their impact on human health, ecosystems, and numerous other domains has become an increasingly prominent field of investigation [3].

Over the past decade, there has been a substantial increase in various omics data types generated from various microbiomes due to the development of novel techniques and reduced experimental costs [4,5]. Hence, the multi-omics approach has emerged as a powerful strategy to elucidate the functional potential of microbiomes, going beyond taxonomic profiling to decipher the molecular mechanisms [6–8]. The metabolic pathways, ecological interactions, and adaptive responses of microbial communities can be uncovered by integrating multiple omics data [9]. Such a comprehensive perspective is invaluable for potential implications in diverse fields, such as human health, agriculture, and environmental conservation.

To unravel the complex web of interactions within microbiomes and extract meaningful insights from the vast amount of data generated by advanced omics technologies, the development of sophisticated analytical tools and data analysis pipelines is essential [10]. Consequently, many approaches and tools have emerged to address these needs [11–15]. One such pipeline, gNOMO, facilitates the integrated multi-omics analysis encompassing metagenomics (MG), metatranscriptomics (MT), and metaproteomics (MP) through the

efficient generation and use of a proteogenomic database, as well as differential abundance analysis-based integration at the pathway and taxa levels [16]. However, gNOMO (along with other existing multi-omics analysis tools in microbiome field) currently lacks the capability of processing 16S rRNA gene amplicon sequencing (AS) data to create a proteogenomic database while it is well known that the protein sequence database directly impacts the outcome of any MP analysis [17].

For MP, it was shown that unnecessarily large databases can lead to the exclusion of valid peptide spectrum matches [18], demanding more time and memory resources. Conversely, smaller databases carry the risk of generating false positive results that are irrelevant to the sample. In multi-omics based microbiome studies that combine MP with MG or MT, protein databases are typically generated from MG and MT data. However, for studies that integrate MP with AS, there is currently no tool available to automatically create a protein database from AS data for MP analysis. Generating an AS-based protein database can also be valuable for studies that integrate MP, MG and MT, as sequencing depth limitations may affect the detection of microbes and genes, thereby influencing MP analysis. Additionally, there is a lack of tools for conducting end-to-end integrated analysis of AS data in conjunction with MP results. Most existing multi-omics analysis tools are tailored to specific omics combinations and lack a modular architecture that can accommodate various omics combinations. Furthermore, there is a shortage of multi-omics analysis tools that incorporate multiple integration approaches and present results at different analysis stages, facilitating further investigations using other tools.

To address abovementioned needs of multi-omics data analyses in microbiome research, we have made significant improvements to the gNOMO pipeline. These enhancements encompass the following key modifications: i) We have restructured the pipeline, introducing a flexible and modular architecture which empowers gNOMO2 to seamlessly process a wide array of

multi-omics data derived from microbiomes. With six independent modules, gNOMO2 can effortlessly manage a vast spectrum of omics combinations, ranging from two to four distinct omics data types, which include AS, MG, MT, and MP. ii) One of standout features of gNOMO2 is its ability to process AS data and generate a protein database suitable for MP studies. iii) Additionally, gNOMO2 incorporates three distinct approaches for integrated multi-omics analysis: proteogenomic database-based integration, differential abundance-based integration (at taxa, functional category and pathway levels), and joint visualization-based integration. These innovative approaches offer a comprehensive perspective on the microbiomes, enabling researchers to gain deeper insights into the structural and functional properties. gNOMO2 is an open-source tool and freely available at https://github.com/muzafferarikan/gNOMO2.

Methods

Overview of the gNOMO2 pipeline

The gNOMO2 pipeline is designed as a tool that relies on Snakemake [19], a well-established bioinformatic workflow management system. This framework guarantees scalable data analyses and the generation of consistent and reproducible output data. The pipeline incorporates a suite of software tools written in various programming languages, including R, Python, Shell and Perl, enabling the seamless execution of multi-omics analysis steps for microbiome data. The input data and program parameters in Snakemake are easily defined through a straightforward configuration file. gNOMO2 streamlines this process by automatically generating the configuration file from the provided input data along with default parameters.

To enhance user experience, the pipeline relies on publicly accessible tools distributed as Conda environments, simplifying the installation process for individual software components for the end user. gNOMO2 ensures result consistency and makes it user-friendly for individuals with basic bioinformatics skills to analyze multi-omics data. The pipeline accepts raw sequencing files (in fastq.gz format) for AS, MG and MT data and input MS/MS spectrum files (in mgf format) for MP data.

The original gNOMO accepts MG, MT and MP data as input and generates results for differential abundance analysis in each omics layer. It also constructs a protein database using MG and MT data and performs both differential abundance and pathway level integrated analyses (Figure 1A). In contrast, gNOMO2 pipeline comprises six modules that facilitate direct analysis of various omics combinations. Each module includes pre-processing, analysis of each omics dataset, data integration and visualization steps (Figure 1B). We implemented changes to both the analysis workflow and pipeline structure. For workflow adjustments, we updated the quality control, merging, assembly, differential abundance, and visualization steps. In the quality control phase, we switched from using PrinSeq to Trimmomatic for cleaning and trimming reads. For read merging, we replaced fastq-join with FLASH to merge paired-end reads. In the assembly step, we transitioned from Ray to metaSPAdes for *de novo* assembly of metagenomic sequences, and from Ray to rnaSPAdes for *de novo* assembly of metatranscriptomic sequences. In the differential abundance analysis step, we replaced LefSe with MaAsLin2. For visualization, we replaced Krona with ggplot2 to analyze taxonomic composition, enabling combined visualization of samples. We also replaced LefSe with MaAsLin2 for visualizing the results of differential abundance analysis. For pathway-level analysis results, we kept Pathview unchanged, but for joint-visualization analysis, we used the combi package to visualize outputs. These workflow changes and comparisons between gNOMO and gNOMO2 are depicted in Supplementary Fig. S1.

Additionally, we introduced changes to facilitate the incorporation of metadata tables into analyses and automated the creation of the configuration file. To enhance and update the structure of the original gNOMO, we implemented six modules in the new gNOMO2 pipeline, allowing for the processing of different omics combinations. The original gNOMO pipeline consisted of only one module (Module 5 in gNOMO2), while gNOMO2 introduced five more modules for specific combinations, along with the ability to accept AS data as input.

Figure 1. Overview of gNOMO and gNOMO2 pipelines. (A) gNOMO accepts MG, MT and MP data as input, providing differential abundance analysis results for each omics layer. It also generates a protein database using MG and MT data and performs a pathway-level integrated analysis. (B) gNOMO2 comprises six modules, each tailored for specific omics data. Module 1 accepts 16S rRNA gene amplicon sequencing data (AS) as input and generates a protein database suitable for metaproteomics studies, a taxa abundance plot and a phyloseq object that can be used for downstream analysis in other microbiome tools. Modules 2 to 6 handle different combinations of AS, MG, MT, and MP data, creating omics-specific protein databases, abundance tables, plots, differential abundance analysis results, and pathway-level integration analysis results.

Module 1: Processing AS data and generating a protein database for MP analysis

Module 1 is designed to process raw AS data in both paired-end and single-end formats, providing a directly usable protein database for MP data analysis. The first step in this module involves using Trimmomatic [20] to remove sequencing adapters, low quality bases from raw reads and reads that are too short (default minimum length > 25 bp). The quality of both raw and trimmed reads is assessed using FastQC [21], and analysis results for all samples are summarized using MultiQC [22]. If the data is in paired-end format, the quality controlled reads are merged using FLASH2 [23]. Subsequently, DADA2 [24] is used in conjunction with the SILVA database [25] to obtain an amplicon sequence variant (ASV) abundance table and taxonomy assignments for each ASV. After determining the user defined top n most abundant

taxa at a user defined taxonomic level, protein sequences of all complete genomes for these taxa are downloaded from the National Center for Biotechnology Information (NCBI) database using the ncbi-genome-download (https://github.com/kblin/ncbi-genome-download) tool. All downloaded sequences are merged and cleaned, and a single protein sequence is retained from identical protein sequences to effectively remove redundancy using SeqKit [26]. Importantly, for host associated microbiome samples, the user can define host species name in a configuration file. Host protein sequences are then included in the final protein database together with microbial proteins. Module 1 allows researchers to construct a comprehensive protein database, either from their own AS datasets or publicly available ones. Furthermore, this module creates a phyloseq [27] object containing an abundance table, a taxonomy table and additional metadata. This enables ongoing microbiome analysis using other analysis tools. In addition, an abundance plot is automatically generated to assess the abundance distribution of the top 'n' taxa as defined by the user.

Module 2: Integrated multi-omics analysis of AS and MP data

Module 2 accepts raw paired-end and single-end AS and MP data as inputs. The AS data undergoes the processing steps described in Module 1. The generated AS-based protein database is then used for the database search algorithm MS-GF+ [28] to identify peptides in the raw MP data. A peptide abundance table is subsequently created by aggregating results from individual samples. Taxonomy and enzyme commission (EC) assignments for the identified peptides are carried out using Pyteomics [29] and Unipept [30]. Then, MaAsLin2 [31] is employed to determine differentially abundant taxa based on both AS and MP data. In this analysis, linear models are employed to identify taxa that exhibit significant differences in abundance between sample groups at AS and MP levels while accounting for confounding variables and other factors that might impact the abundance of microbial taxa. Users can define

the phenotype of interest, covariates in the Snakemake configuration file. Furthermore, users can specify the normalization or transformation to apply prior to conducting the differential abundance analysis. Furthermore, a joint visualization of MP and AS results is performed using the combi R package [32]. This joint visualization allows to integrate and compare the results from both types of omics data (taxa for AS and peptides for MP), providing a comprehensive view on a single ordination plot and helping researchers to identify associations of features from different omics datasets and covariates in metadata table. The final outputs include abundance tables based on both AS and MP data, detailing the abundance of taxa and peptides in each sample, respectively. Module 1 also generates results from the differential abundance analysis, highlighting the taxa that were significantly different between sample groups based on their AS and MP profiles and the joint visualization analysis results providing a graphical representation of the combined AS and MP features, aiding in the interpretation of the integrated results.

Module 3: Integrated multi-omics analysis of MG and MP data

Module 3 is designed to handle raw paired-end MG and MP data. MP data is processed as outlined in Module 2. MG raw reads are quality checked and cleaned using Trimmomatic [20], followed by merging with FLASH [23]. The quality of both raw and trimmed reads is assessed using FastQC [21], and analysis results for all samples are summarized using MultiQC [22]. Cleaned and merged reads are then mapped to the NCBI non-redundant (nr) database using Kaiju [33], which generates taxonomic classification results. In parallel, clean reads are also used for assembly with metaSPAdes [34] and obtained contigs are classified as eukaryotic and prokaryotic using EukRep [35]. Proteins within the prokaryotic contigs are predicted using Prodigal [36] while Augustus [37] is used for proteins within eukaryotic contigs. Then, functional annotation of these predicted proteins is carried out using EggNOG [38] to obtain

KEGG Orthology (KO) identifiers, while InterProScan [39] is employed for TIGRFAM [40] functional annotation.

Module 3 generates several final outputs for both MG and MP analyses. These include taxonomic abundance tables, taxonomic composition plots, and results from taxa and functional annotation (TIGRFAM)-based differential abundance analyses. The module also provides integrated analysis outputs: i) a joint visualization of omics layers as described in Module 2 and ii) a pathway-level integrated analysis is conducted using the Pathview [41] package. The Pathview plots in this analysis illustrate the log2 ratio of the mean abundance of individual omic features under different user-defined conditions across various omics levels, following a fold change normalization. These log2 ratios are calculated and compared using shared enzyme and KEGG ids between different omics layers. Coverages for gene sequences of each predicted protein by MG are calculated using BBMap [42]. The calculated ratios are visualized on metabolic pathway nodes, which are split into omics types (for example, two splits for Module 3 for MG and MP data). The color of each split part shows the abundance change in the relevant features between sample groups for the specific omics level, allowing for the visual tracking of changes in different omics levels on the same node.

Module 4: Integrated multi-omics analysis of MG and MT data

Module 4 is designed to handle raw paired-end MG and both paired-end and single-end MT data. MG data follows the processing steps outlined in Module 3. For MT data, a similar workflow is employed, with the exception that a *de novo* assembly step is conducted using rnaSPAdes [43] in place of metaSPAdes. The final outputs of Module 4 include an MG&MT-based proteogenomic database, taxonomic and functional annotation based differential abundance analysis results for both omics levels, taxonomic abundance tables and plots, joint

visualization of omics layers as described in Module 2 and pathway-level integrated analysis results as outlined in Module 3.

Module 5: Integrated multi-omics analysis of MG, MT and MP data

Module 5 accepts raw paired end MG, both paired-end and single-end MT and MP data. MG and MT data follow the processing steps outlined in Module 4 while MP data is processed as described in Module 2. The final outputs of Module 5 include a MG&MT-based proteogenomic database, taxonomic and functional annotation based differential abundance analysis results for three omics levels, taxonomic abundance tables and plots, peptide abundance table for MP, joint visualization of omics layers and pathway level integrated analysis results as outlined in Module 3.

Module 6: Integrated multi-omics analysis of AS, MG, MT and MP data

Module 6 accepts both paired-end and single-end AS and MT data, paired end MG, and MP data. MG, MT and MP data follow the processing steps outlined in Module 5. However, the final outputs of Module 6 include a proteogenomic database which is generated by combining AS, MG and MT based downloaded/predicted protein sequences, taxonomic and functional annotation based differential abundance analysis results for four omics levels, taxonomic/peptide abundance tables and plots, joint visualization of omics layers and pathway level integrated analysis results as outlined in Module 3.

Analyses

To illustrate the utility of gNOMO2, we re-analyzed samples from four previously published microbiome studies involving various multi-omics combinations, using the respective publicly available datasets.

Analyzing the association of saliva content with oral cancer

Saliva is a complex biofluid that comprises various components, including DNA, RNA, proteins, metabolites, and microbiota. As a result, it is considered as a promising source of relevant biomarkers for a variety of diseases [44]. Granato *et al.* (2021) combined AS and MP analyses to investigate the association between saliva content and oral cancer [45]. The study suggests that oral microbiota and their protein abundance have potential diagnosis and prognosis value for oral cancer patients. Here, we showcase how Modules 1 (AS) 2 (AS and MP) of gNOMO2 can be used to efficiently reproduce the findings.

The AS data was obtained from NCBI SRA under BioProject identifier PRJNA700849 while MP data was retrieved from PRIDE under accession number PXD022859. The dataset included saliva samples from 8 healthy controls and 15 oral cancer patients. To streamline downstream analyses, we merged triplicates of AS samples and used cell debris MP samples for all analyses. The taxonomic composition results based on AS data across samples, as generated by gNOMO2, were consistent with the reported results, demonstrating similar abundance distributions and the presence of the same most abundant genera (Figure 2A). In their study, Granato *et al.* (2021) constructed a protein database containing 1,160,275 protein sequences from the 12 most abundant bacterial genera and human. We applied the same parameters in gNOMO2 to achieve comparable results, with setting such as taxa_level: Genus, top_n: 12 and host: Homo sapiens. gNOMO2 automatically generated a protein database from AS data by determining the 12 most abundant bacterial genera. It then retrieved all protein

sequences from 1,992 genomes belonging these bacterial genera, along with human host proteins, resulting in a total of 1,240,988 protein sequences. The discrepancy in the number of protein sequences between the generated protein databases may be attributed to variations in analysis timing and database differences. Granato *et al.* (2021) used the HOMD, a specific database used for oral microbiome studies while gNOMO2 uses the NCBI database, intended to target all microbiome study types.

Within gNOMO2 users can also perform differential abundance analysis at both omics levels, yielding statistical test results and plots for differential abundant taxa. For instance, we presented one of differential taxa from AS-based (Figure 2B, upper) and MP-based results (Figure 2B, lower). AS-based differential abundance analysis showed a decrease in the abundance of *Veillonella* associated with oral cancer (Figure 2B, upper), which corresponds to a key finding in the Granato *et al.* (2021) study and previous studies [46]. Interestingly, gNOMO2 detected a reduction in the abundance of peptides classified as Homo in oral cancer patients (Figure 2B, lower) while the original study did not report any statistically significant changes. This divergence may result from differences in analysis approaches, as gNOMO2 employs a peptide-based taxonomy by Unipept and MaAslin2 for differential abundance analysis instead of a protein-based approach. Furthermore, it is important to note that we did not account for other covariates that may affect the results.

Finally, gNOMO2 generates a joint visualization plot for AS, MP and metadata (Figure 2C). This plot confirms the association of *Veillonella* based on AS with oral health status based on AS data and additionally reveals associations between some detected peptides and the oral health status of the participants. Notably, InterPro entries assigned to these peptides included human albumin proteins, which were previously reported to be associated with oral cancer [47,48].

Figure 2. Overview of gNOMO2 results for the Granato *et al.* (2021) study. (A) Representation of the ten most prevalent genera in saliva microbiota samples. AS-based representations of salivary microbiota composition across samples, highlighting the ten most common bacterial genera. Each bar indicates the relative abundance distribution for a sample. (B) Abundance distribution of differentially abundant taxa across study groups, presented separately for AS (upper) and MP (lower) data. (C) Joint visualization-based integration results for AS, MP and metadata. Blue labels represent taxa, green labels represent peptides, and black labels represent metadata columns. Patient samples are marked with blue dots, while healthy samples are marked with red dots.

Exploring potential and active functions within the human gut microbiota

The human gut microbiota is widely recognized for its important roles in both health and disease. A comprehensive understanding of both potential and active features can provide valuable insights into the mechanisms governing various physiological processes and pathologies, ultimately leading to more effective strategies for maintaining and improving human well-being.

Tanca *et al.* (2017) employed MG and MP to explore the potential and active functions in the gut microbiota of a healthy human cohort [49]. Here, we used Module 3 (MG and MP) of gNOMO2 to efficiently re-analyze the multi-omics data from their study. The MG data was obtained from the NCBI SRA under BioProject identifier PRJEB19090, while the MP data was retrieved from PRIDE under accession number PXD005780. The dataset included gut microbiota samples from 6 males and 8 females.

We employed gNOMO2 to investigate potential differences between male and female participants. Taxonomic composition results based on MG and MP data, as generated by gNOMO2, exhibited a significant overlap with the findings of Tanca *et al.* (2017), particularly concerning the most abundant genera (Figure 3A, upper). MG-based differential abundance analysis, using default parameters, indicated a notably higher abundance of *Legionella* in females. Nevertheless, it is important to approach this finding with caution, given that

Legionella is a bacterial genus typically associated with water and soil environments, often considered a potential source of contamination in human microbiome studies [50].

Functional annotations derived from TIGRFAM for the differential abundance analysis indicated a reduction in biotin synthesis (Figure 3B, lower). The joint visualization plot depicted both MG and MP features along with covariates such as BMI, age and sex (Figure 3C). In our pathway-level integration analysis, we illustrated the components of pyrimidine metabolism and how variations in their abundance can be observed among study groups across different omics levels (Figure 3D). As a case in point, cytidine deaminase (EC 3.5.4.5) displayed a decreased abundance in females at the MG level (colored green, left), while its abundance increased at the MP level (colored red, right). This discrepancy suggests a decrease in the abundance of taxa carrying the corresponding gene but a higher expression of the protein. Again, this highlights the significance of adopting a multi-omics perspective when drawing conclusions in microbiome studies.

Figure 3. Overview of gNOMO2 results for Tanca *et al.* (2017) study. (A) Representation of the ten most prevalent genera in gut microbiota samples, as shown by MG and MP. The left side illustrates the ten most common bacterial genera based on MG data, while the right side represents MP-based findings. Each bar represents relative abundance distribution for a sample. (B) Abundance distribution of differentially abundant taxa across study groups, separately for MG (upper) and MP (lower) data. (C) Joint visualization-based integration results for MG, MP and metadata. Blue labels represent taxa, green labels show peptides and black labels represent metadata columns. Male samples are marked with blue dots, while female samples are marked with red dots. (D) Pathway level integration results, demonstrating the relationship across different omics levels. The findings from MG and MP are illustrated separately as split nodes on the left and right, respectively.

Investigating the role of microbiota of the Maasdam cheese during ripening

The microbiota present in cheese plays a crucial role in the maturation and development of its distinctive flavor, making it a pivotal aspect for the cheese industry. Duru *et al.* (2018)

combined MG and MT to track shifts in both taxonomic compositions and gene expressions of Swiss-type Maasdam cheese microbiota during the ripening process [51]. Here, we used Module 4 (MG and MT) of gNOMO2 to efficiently re-analyze multi-omics data from their research.

MG and MT data were retrieved from the EBI ENA under BioProject identifier PRJEB23938. The dataset comprised three samples from day 12 and three samples from day 37 day of the ripening process.

We employed gNOMO2 to investigate potential differences between different stages of ripening process. Taxonomic composition results generated by gNOMO2 based on MG and MT data showed that *Lactococcus*, *Lactobacillus* and *Propionibacterium* were three most abundant genera across samples (Figure 4A), in consistent with the findings of Duru *et al.* (2018). Differential abundance analyses revealed significantly higher relative abundance of *Propionibacterium*, the main bacterial genera responsible for propionate metabolism in the Maasdam cheese, in cold ripening samples in both MG and MT levels (Figure 4B) which is also well aligning with the results of the original study.

The joint visualization plot depicted both MG and MT features along with the ripening types (Figure 4C). In our exploration of pathway-level integration, we depicted the elements of propionate metabolism and highlighted how fluctuations in their abundance varied across study groups at MG and MT levels (Figure 4D). Notably, genes related to propionate production exhibited higher abundance in cold ripening samples (day 37) compared to warm ripening ones (day 12) at the MT level (colored red, right), while their levels were not significantly different at the MG level (colored gray, left). As a result, we did not observe a decrease in expression of genes responsible for propionate production, contrary to findings in the original study. This discrepancy may originate from methodological differences between the studies. The gNOMO2 pipeline compares the expression of propionate production genes against total gene

expression, whereas Duru *et al.* (2018) study compared these genes against the overall expression of the *Propionibacterium* genome obtained in their research. Consequently, the relative expression of these genes might appear higher when assessed against all genes but lower when measured against only *Propionibacterium* genes. To validate this, we conducted comparisons using the *Propionibacterium* genome from the original study in the gNOMO2 pipeline for gene expression levels. Changing the denominator from all genes to *Propionibacterium* genes yielded results consistent with the original study.

Our findings emphasize the critical role of accurately interpreting analysis outcomes based on the structure of the analytical pipeline. Assuming a default approach, particularly during comparison steps, could lead to unsupported conclusions. In meta-omics studies, various approaches can be employed for data analysis. While none of these approaches are inherently wrong, they may not align with the goals set by the research group. When the pipeline's structure is well-defined, no inconsistencies in biological conclusions would be expected. Additionally, we stress the importance of clear language in explaining results in research articles, as failure to do so may mislead readers. In this instance, the discrepancy was primarily due to differences between the approach depending on comparisons at individual MAG level and the gNOMO2 approach, which compares with the whole community.

Figure 4. Overview of gNOMO2 results for the Duru *et al.* (2018) study. (A) Representation of the 10 most common genera in cheese microbiota samples. MG- and MT-based overview of gut microbiota composition across samples. The 10 most common bacterial genera in cheese microbiota samples are shown for MG (left) and MT (right). Each bar represents relative abundance distribution for a sample. (B) Abundance distribution of differentially abundant taxa across study groups by MG (upper) and MT (lower). (C) Joint visualization-based integration results for MG, MT and metadata. (D) Pathway level integration results, demonstrating the relationship across different omics levels. The findings from MG and MT are illustrated separately as split nodes on the left and right, respectively.

Determining microbiome dynamics in a wastewater treatment plant

Characterization of microbial communities across various meta-omics layers offers important insights into their potential traits and functionalities. Herold et al. (2020) utilized MG, MT, MP, and metabolomics to explore the responses of microbial populations in a biological wastewater treatment plant to disturbances. In our study, we demonstrate how Modules 5 (MG, MT, and MP) and 6 (AS, MG, MT, and MP) of gNOMO2 effectively replicate some of their findings using a subset of the samples.

We obtained AS, MG, and MT sequencing data from EBI ENA (BioProject identifier PRJNA230567) and MP data from PRIDE (accession number PXD013655). To investigate seasonal variations reported by Herold et al. (2020), we selected samples showcasing the most distinct differences between summer and winter seasons, encompassing five samples from each. Additionally, we incorporated 10 AS samples previously collected from the same wastewater treatment plant by the same research group to assess Module 6.

Our analysis, performed using gNOMO2, revealed taxonomic composition results (AS, MG, MT, and MP data) that partially aligned with Herold et al.'s findings (Figure 5A). However, unlike the original study, we did not observe pronounced compositional changes in winter samples (Figure 5A). This discrepancy may be attributed to differing approaches in taxonomic composition analysis as Herold et al. utilized taxonomic assignments of a subset of metagenome assembled genomes, while gNOMO2 employs Kaiju for direct taxonomic classification of reads.

While gNOMO2 did not detect differentially abundant taxa between seasons across MG, MT, and MP layers, our TIGRFAM and KEGG pathway-based analyses indicated an elevation in fatty acid degradation at the MT level (Figure 5B), aligning with the original study. The joint-visualization plot highlighted MG, MT and MP features along with covariates (Figure 5C). As a case point, the plot revealed the association of *Tetrasphaera* with autumn which has

been reported in previous studies to be associated with sludge bulking that frequently occurs in wastewater treatment plants [52,53].

In our pathway-level integration analysis (Figure 5D), we illustrated variations in the components of fatty acid degradation and glycerolipid metabolism among study groups across different omics levels. Specifically, gNOMO2 showcased an increase in fatty acid degradation at the MT level while detecting an elevation in glycerolipid metabolism at both MT and MP levels, as indicated and discussed in detail in the original paper.

When AS data were integrated using Module 6, gNOMO2 constructed a proteogenomic database comprising 4,959,677 proteins, incorporating 859,729 non-redundant proteins derived from the top 10 most abundant genera identified in the AS analysis, in addition to the 4,025,111 proteins obtained from MG and MT analyses. Interestingly, this integration resulted a slight decrease in the number of detected unique peptides (~2%), indicating the importance of database size optimization in the multi-omics studies including MP. The inclusion of AS data did not alter the other outcomes derived from the MP data analysis.

Figure 5. Overview of gNOMO2 results for the Herold *et al.* (2020) study. (A) Representation of the 10 most common genera in wastewater microbiota samples. MG-, MT- and MP-based overview of gut microbiota composition across samples. The 10 most common bacterial genera in wastewater microbiota samples by MG (left), MT (middle) and MP (right). Each bar represents relative abundance distribution for a sample. (B) Abundance distribution of differentially abundant taxa across study groups by MG (upper), MT (middle) and MP (lower). (C) Joint visualization-based integration results for MG, MT, MP and metadata. (D) Pathway level integration results, demonstrating the relationship across different omics levels. The findings from MG, MT, and MP are illustrated separately as split nodes on the left, middle, and right, respectively.

Our findings highlight that read-based and MAG-based taxonomic composition analysis approaches can lead to divergent results and interpretations. Since neither approach is inherently wrong, this disparity underscores the significance and advantage of thoroughly

examining meta-omics datasets using various methodologies. Hence, we underscore that employing diverse approaches and perspectives in complex multi-omics datasets may reveal novel insights extending beyond the original hypothesis.

Discussion

gNOMO2 stands as a versatile and modular bioinformatic pipeline designed for integrated multi-omics analyses of AS, MG, MT, and MP data in a reproducible fashion. Our open-source tool efficiently employs techniques that process raw data and generates summary tables and figures with just a single, straightforward command. gNOMO2 encompasses preprocessing, genome mapping, assembly, protein predictions, taxonomic and functional annotations, proteogenomic database generation and differential abundance analysis steps for each omics layer. Furthermore, gNOMO2 offers a holistic perspective through integrated visualization of omics layers and facilitates pathway-level integrative analysis. In addition, it includes a dedicated module for AS data processing and the automatic protein database generation for MP studies. gNOMO2 generates results that can serve as inputs for subsequent microbiome analyses using various bioinformatics tools, enhancing user flexibility throughout the process. Demonstrated efficacy of gNOMO2 with real datasets underscores it as an invaluable tool across various multi-omics combinations in microbiome research. Finally, the emphasis on reproducibility is a cornerstone of gNOMO2, as it not only streamlines the analytical process but also ensures the reliability of results by providing users with fully documented and executable workflows, enhancing the transparency and replicability in omics-driven microbiome research.

Despite its usefulness and effectiveness in multi-omics based microbiome research, gNOMO2 still has certain limitations. Firstly, its performance may be influenced by the quality

and depth of input data, thereby necessitating potential parameter optimizations by the user. Secondly, gNOMO2 relies on existing databases for taxonomic and functional annotations which may restrict the detection of features not cataloged within these databases. Moreover, gNOMO2's efficacy may also be influenced by the complexity of microbial communities, particularly in cases of high diversity or rare taxa, where accurate profiling may be challenging. Lastly, users should be aware that gNOMO2 assumes a certain level of computational proficiency, and while efforts have been made to enhance user-friendliness, beginners may still face a learning curve because there is no graphical user interface provided.

Future versions of gNOMO2 could address these limitations through continuous updates, improved algorithmic approaches, and increased flexibility in handling diverse omics types, datasets and microbial community structures.

Availability of source code and requirements

Project name: gNOMO2

Project home page: https://github.com/muzafferarikan/gNOMO2

Operating system(s): GNU/Linux

Programming language: Python, R, Shell and Perl

Other requirements: Conda and Snakemake are required for implementation. At least 1 TB hard drive space and 200 GB memory are recommended to run the pipeline, dependent on databases and input file sizes used.

License: MIT

Restrictions to use by non-academics: No

RRID: SCR_025293

BioTools ID: gnomo2

Abbreviations

NCBI: National Center for Biotechnology Information, ENA: European Nucleotide Archive, MIT: Massachusetts Institute of Technology, SRA: Sequence Read Archive.

Acknowledgements

Muzaffer Arıkan is a recipient of the Scientific and Technological Research Council of Turkey (TUBITAK), BIDEB 2219-International Postdoctoral Research Fellowship.

Authors' contributions

MA and TM conceived the idea and designed the pipeline. MA implemented the pipeline, performed analyses and wrote the manuscript. TM reviewed and edited the manuscript. All authors approved the final version of the manuscript.

Competing interests

The authors declare that they have no competing interests.

Ethical Approval

Not applicable.

Funding

This work was supported through funding from the Scientific and Technological Research Council of Turkey (TUBITAK), BIDEB 2219-International Postdoctoral Research Fellowship granted to Muzaffer Arıkan.

Availability of data and materials

The datasets supporting the conclusions of this article are available in the NCBI SRA and PRIDE databases. Sequencing datasets can be searched under the following NCBI BioProject identifiers: PRJNA700849 [45], PRJEB19090 [49], PRJEB23938 [51], PRJNA230567 [54]. Metaproteomics datasets can be searched under the following PRIDE accession numbers: PXD022859 [45], PXD013655 [54].

The gNOMO2 software is freely available under the MIT license and can be accessed through https://github.com/muzafferarikan/gNOMO2.

References

1. Ogunrinola GA, Oyewale JO, Oshamika OO, Olasehinde GI. The Human Microbiome and Its Impacts on Health. *Int J Microbiol*. 2020; doi: 10.1155/2020/8045646.

2. Blaser MJ, Cardon ZG, Cho MK, Dangl JL, Donohue TJ, Green JL, et al.. Toward a Predictive Understanding of Earth's Microbiomes to Address 21st Century Challenges. *MBio*. 2016; doi: 10.1128/mBio.00714-16.

3. Berg G, Rybakova D, Fischer D, Cernava T, Vergès M-CC, Charles T, et al.. Microbiome definition re-visited: old concepts and new challenges. *Microbiome*. Microbiome; 2020; doi: 10.1186/s40168-020-00875-0.

4. Zhang X, Li L, Butcher J, Stintzi A, Figeys D. Advancing functional and translational microbiome research using meta-omics approaches. *Microbiome*. Microbiome; 2019; doi: 10.1186/s40168-019-0767-6.

5. Ari Ş, Arikan M. Next-Generation Sequencing: Advantages, Disadvantages, and Future. *Plant Omi Trends Appl*. Cham: Springer International Publishing;

6. Daliri EB-M, Ofosu FK, Chelliah R, Lee BH, Oh D-H. Challenges and Perspective in

Integrated Multi-Omics in Gut Microbiota Studies. *Biomolecules*. 2021; doi: 10.3390/biom11020300.

7. Ferrocino I, Rantsiou K, McClure R, Kostic T, de Souza RSC, Lange L, et al.. The need for an integrated multi-OMICs approach in microbiome science in the food system. *Compr Rev Food Sci Food Saf*. 2023; doi: 10.1111/1541-4337.13103.

8. Zhang N, Kandalai S, Zhou X, Hossain F, Zheng Q. Applying multi-omics toward tumor microbiome research. *iMeta*. 2023; doi: 10.1002/imt2.73.

9. Arıkan M, Muth T. Integrated multi-omics analyses of microbial communities: a review of the current state and future directions. *Mol Omi*. 2023; doi: 10.1039/D3MO00089C.

10. Bharti R, Grimm DG. Current challenges and best-practice protocols for microbiome analysis. *Brief Bioinform*. 2021; doi: 10.1093/bib/bbz155.

11. Narayanasamy S, Jarosz Y, Muller EEL, Heintz-Buschart A, Herold M, Kaysen A, et al.. IMP: a pipeline for reproducible reference-independent integrated metagenomic and metatranscriptomic analyses. *Genome Biol*. Genome Biology; 2016; doi: 10.1186/s13059-016-1116-8.

12. Singh A, Shannon CP, Gautier B, Rohart F, Vacher M, Tebbutt SJ, et al.. DIABLO: An integrative approach for identifying key molecular drivers from multi-omics assays. *Bioinformatics*. 2019; doi: 10.1093/bioinformatics/bty1054.

13. Argelaguet R, Velten B, Arnol D, Dietrich S, Zenz T, Marioni JC, et al.. Multi-Omics Factor Analysis—a framework for unsupervised integration of multi-omics data sets. *Mol Syst Biol*. 2018; doi: 10.15252/msb.20178124.

14. Eren AM, Esen OC, Quince C, Vineis JH, Morrison HG, Sogin ML, et al.. Anvi'o: An advanced analysis and visualization platformfor 'omics data. *PeerJ*. 2015; doi: 10.7717/peerj.1319.

15. Bolyen E, Rideout JR, Dillon MR, Bokulich NA, Abnet CC, Al-Ghalith GA, et al..

Reproducible, interactive, scalable and extensible microbiome data science using QIIME 2. *Nat Biotechnol*. 2019; doi: 10.1038/s41587-019-0209-9.

16. Muñoz-Benavent M, Hartkopf F, Van Den Bossche T, Piro VC, García-Ferris C, Latorre A, et al.. gNOMO: a multi-omics pipeline for integrated host and microbiome analysis of non-model organisms. *NAR Genomics Bioinforma*. 2020; doi: 10.1093/nargab/lqaa058.

17. Blakeley-Ruiz JA, Kleiner M. Considerations for constructing a protein sequence database for metaproteomics. *Comput Struct Biotechnol J*. The Authors; 2022; doi: 10.1016/j.csbj.2022.01.018.

18. Muth T, Kolmeder CA, Salojärvi J, Keskitalo S, Varjosalo M, Verdam FJ, et al.. Navigating through metaproteomics data: a logbook of database searching. *Proteomics*. 2015; doi: 10.1002/pmic.201400560.

19. Koster J, Rahmann S. Snakemake--a scalable bioinformatics workflow engine. *Bioinformatics*. 2012; doi: 10.1093/bioinformatics/bts480.

20. Bolger AM, Lohse M, Usadel B. Trimmomatic: a flexible trimmer for Illumina sequence data. *Bioinformatics*. 2014; doi: 10.1093/bioinformatics/btu170.

21. Arıkan M, Demir TK, Yıldız Z, Nalbantoğlu ÖU, Korkmaz ND, Yılmaz NH, et al.. Metaproteogenomic analysis of saliva samples from Parkinson’s disease patients with cognitive impairment. *npj Biofilms Microbiomes*. 2023; doi: 10.1038/s41522-023-00452-x.

22. Ewels P, Magnusson M, Lundin S, Käller M. MultiQC: summarize analysis results for multiple tools and samples in a single report. *Bioinformatics*. 2016; doi: 10.1093/bioinformatics/btw354.

23. Magoc T, Salzberg SL. FLASH: fast length adjustment of short reads to improve genome assemblies. *Bioinformatics*. 2011; doi: 10.1093/bioinformatics/btr507.

24. Callahan BJ, McMurdie PJ, Rosen MJ, Han AW, Johnson AJA, Holmes SP. DADA2: High-resolution sample inference from Illumina amplicon data. *Nat Methods*. 2016; doi:

10.1038/nmeth.3869.

25. Quast C, Pruesse E, Yilmaz P, Gerken J, Schweer T, Yarza P, et al.. The SILVA ribosomal RNA gene database project: Improved data processing and web-based tools. *Nucleic Acids Res*. 2013; doi: 10.1093/nar/gks1219.

26. Shen W, Le S, Li Y, Hu F. SeqKit: A cross-platform and ultrafast toolkit for FASTA/Q file manipulation. *PLoS One*. 2016; doi: 10.1371/journal.pone.0163962.

27. McMurdie PJ, Holmes S. phyloseq: an R package for reproducible interactive analysis and graphics of microbiome census data. Watson M, editor. *PLoS One*. 2013; doi: 10.1371/journal.pone.0061217.

28. Kim S, Pevzner PA. MS-GF+ makes progress towards a universal database search tool for proteomics. *Nat Commun*. Nature Publishing Group; 2014; doi: 10.1038/ncomms6277.

29. Levitsky LI, Klein JA, Ivanov M V., Gorshkov M V.. Pyteomics 4.0: Five Years of Development of a Python Proteomics Framework. *J Proteome Res*. 2019; doi: 10.1021/acs.jproteome.8b00717.

30. Gurdeep Singh R, Tanca A, Palomba A, Van der Jeugt F, Verschaffelt P, Uzzau S, et al.. Unipept 4.0: Functional Analysis of Metaproteome Data. *J Proteome Res*. 2019; doi: 10.1021/acs.jproteome.8b00716.

31. Mallick H, Rahnavard A, McIver LJ, Ma S, Zhang Y, Nguyen LH, et al.. Multivariable association discovery in population-scale meta-omics studies. Coelho LP, editor. *PLoS Comput Biol*. 2021; doi: 10.1371/journal.pcbi.1009442.

32. Hawinkel S, Bijnens L, Cao K-AL, Thas O. Model-based joint visualization of multiple compositional omics datasets. *NAR Genomics Bioinforma*. Oxford University Press; 2020; doi: 10.1093/nargab/lqaa050.

33. Menzel P, Ng KL, Krogh A. Fast and sensitive taxonomic classification for metagenomics with Kaiju. *Nat Commun*. Nature Publishing Group; 2016; doi: 10.1038/ncomms11257.

34. Nurk S, Meleshko D, Korobeynikov A, Pevzner PA. metaSPAdes: a new versatile metagenomic assembler. *Genome Res*. 2017; doi: 10.1101/gr.213959.116.

35. West PT, Probst AJ, Grigoriev I V., Thomas BC, Banfield JF. Genome-reconstruction for eukaryotes from complex natural microbial communities. *Genome Res*. 2018; doi: 10.1101/gr.228429.117.

36. Hyatt D, Chen G-L, LoCascio PF, Land ML, Larimer FW, Hauser LJ. Prodigal: prokaryotic gene recognition and translation initiation site identification. *BMC Bioinformatics*. 2010; doi: 10.1186/1471-2105-11-119.

37. Stanke M, Morgenstern B. AUGUSTUS: a web server for gene prediction in eukaryotes that allows user-defined constraints. *Nucleic Acids Res*. 2005; doi: 10.1093/nar/gki458.

38. Huerta-Cepas J, Szklarczyk D, Heller D, Hernández-Plaza A, Forslund SK, Cook H, et al.. eggNOG 5.0: a hierarchical, functionally and phylogenetically annotated orthology resource based on 5090 organisms and 2502 viruses. *Nucleic Acids Res*. 2019; doi: 10.1093/nar/gky1085.

39. Jones P, Binns D, Chang H-Y, Fraser M, Li W, McAnulla C, et al.. InterProScan 5: genome-scale protein function classification. *Bioinformatics*. 2014; doi: 10.1093/bioinformatics/btu031.

40. Haft DH, Selengut JD, Richter RA, Harkins D, Basu MK, Beck E. TIGRFAMs and Genome Properties in 2013. *Nucleic Acids Res*. 2013; doi: 10.1093/nar/gks1234.

41. Luo W, Brouwer C. Pathview: an R/Bioconductor package for pathway-based data integration and visualization. *Bioinformatics*. 2013; doi: 10.1093/bioinformatics/btt285.

42. Bushnell B. BBMap: a fast, accurate, splice-aware aligner. *Lawrence Berkeley Natl Lab(LBNL), Berkeley, CA (United States)*.

43. Bushmanova E, Antipov D, Lapidus A, Prjibelski AD. rnaSPAdes: a de novo transcriptome assembler and its application to RNA-Seq data. *Gigascience*. Oxford University Press; 2019;

doi: 10.1093/gigascience/giz100.

44. Aro K, Wei F, Wong DT, Tu M. Saliva Liquid Biopsy for Point-of-Care Applications. *Front Public Heal*. 2017; doi: 10.3389/fpubh.2017.00077.

45. Granato DC, Neves LX, Trino LD, Carnielli CM, Lopes AFB, Yokoo S, et al.. Meta-omics analysis indicates the saliva microbiome and its proteins associated with the prognosis of oral cancer patients. *Biochim Biophys Acta - Proteins Proteomics*. 2021; doi: 10.1016/j.bbapap.2021.140659.

46. Chang X, Chen Y, Cui D, Wang Y, Zhou Y, Zhang X, et al.. Propionate-producing Veillonella parvula regulates the malignant properties of tumor cells of OSCC. *Med Oncol*. Springer US; 2023; doi: 10.1007/s12032-023-01962-6.

47. Shpitzer T, Bahar G, Feinmesser R, Nagler RM. A comprehensive salivary analysis for oral cancer diagnosis. *J Cancer Res Clin Oncol*. 2007; doi: 10.1007/s00432-007-0207-z.

48. Mu AK-W, Chan YS, Kang SS, Azman SN, Zain RB, Chai WL, et al.. Detection of host-specific immunogenic proteins in the saliva of patients with oral squamous cell carcinoma. *J Immunoassay Immunochem*. 2014; doi: 10.1080/15321819.2013.836535.

49. Tanca A, Abbondio M, Palomba A, Fraumene C, Manghina V, Cucca F, et al.. Potential and active functions in the gut microbiota of a healthy human cohort. *Microbiome*. Microbiome; 2017; doi: 10.1186/s40168-017-0293-3.

50. Milani C, Duranti S, Bottacini F, Casey E, Turroni F, Mahony J, et al.. The first microbial colonizers of the human gut: composition, activities, and health Implications of the infant gut microbiota. *Microbiol Mol Biol Rev*. 2017; doi: 10.1128/MMBR.00036-17.

51. Duru IC, Laine P, Andreevskaya M, Paulin L, Kananen S, Tynkkynen S, et al.. Metagenomic and metatranscriptomic analysis of the microbial community in Swiss-type Maasdam cheese during ripening. *Int J Food Microbiol*. Elsevier; 2018; doi: 10.1016/j.ijfoodmicro.2018.05.017.

52. Wang P, Yu Z, Qi R, Zhang H. Detailed comparison of bacterial communities during seasonal sludge bulking in a municipal wastewater treatment plant. *Water Res*. Elsevier Ltd; 2016; doi: 10.1016/j.watres.2016.08.050.

53. Xu S, Yao J, Ainiwaer M, Hong Y, Zhang Y. Analysis of Bacterial Community Structure of Activated Sludge from Wastewater Treatment Plants in Winter. *Biomed Res Int*. 2018; doi: 10.1155/2018/8278970.

54. Herold M, Martínez Arbas S, Narayanasamy S, Sheik AR, Kleine-Borgmann LAK, Lebrun LA, et al.. Integration of time-series meta-omics data reveals how microbial ecosystems respond to disturbance. *Nat Commun*. Springer US; 2020; doi: 10.1038/s41467-020-19006-2.

Figure 1

Click here to access/download;Figure;Figure_1.pdf

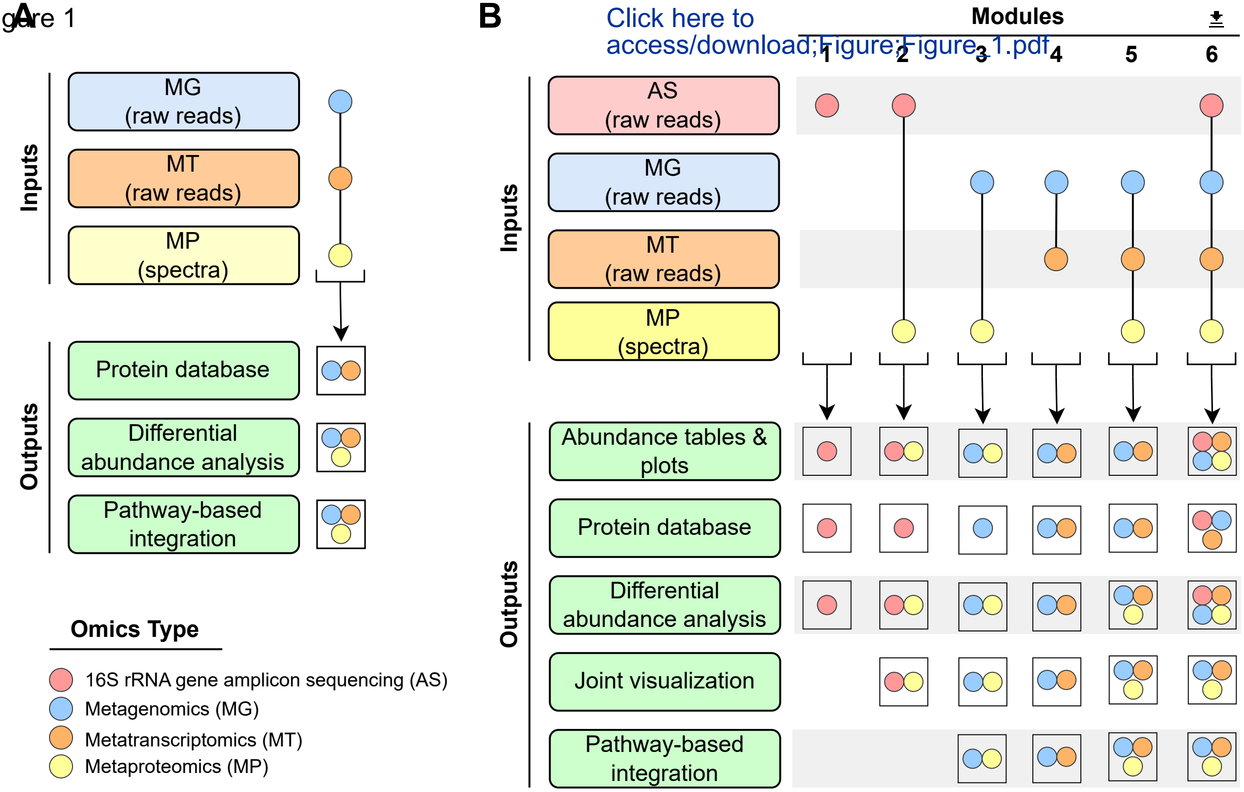

Figure 2

Click here to access/download;Figure;Figure_2.pdf

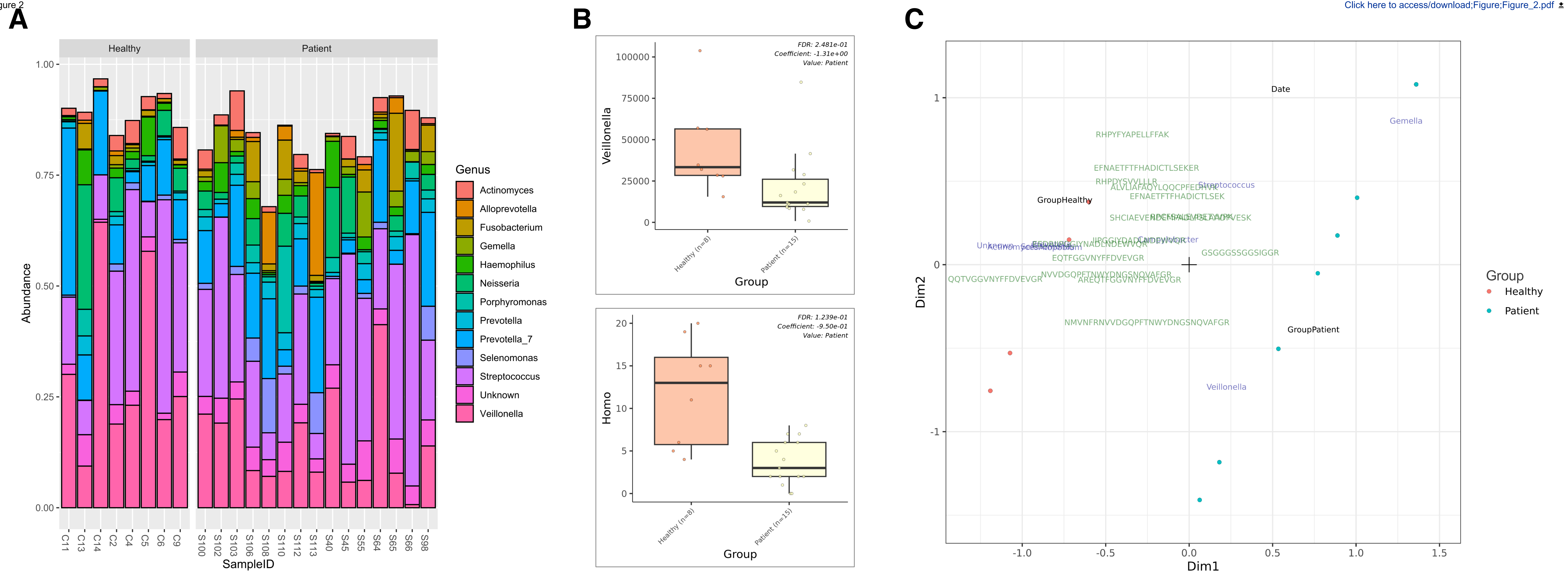

Figure 3

Click here to access/download;Figure;Figure_3.pdf

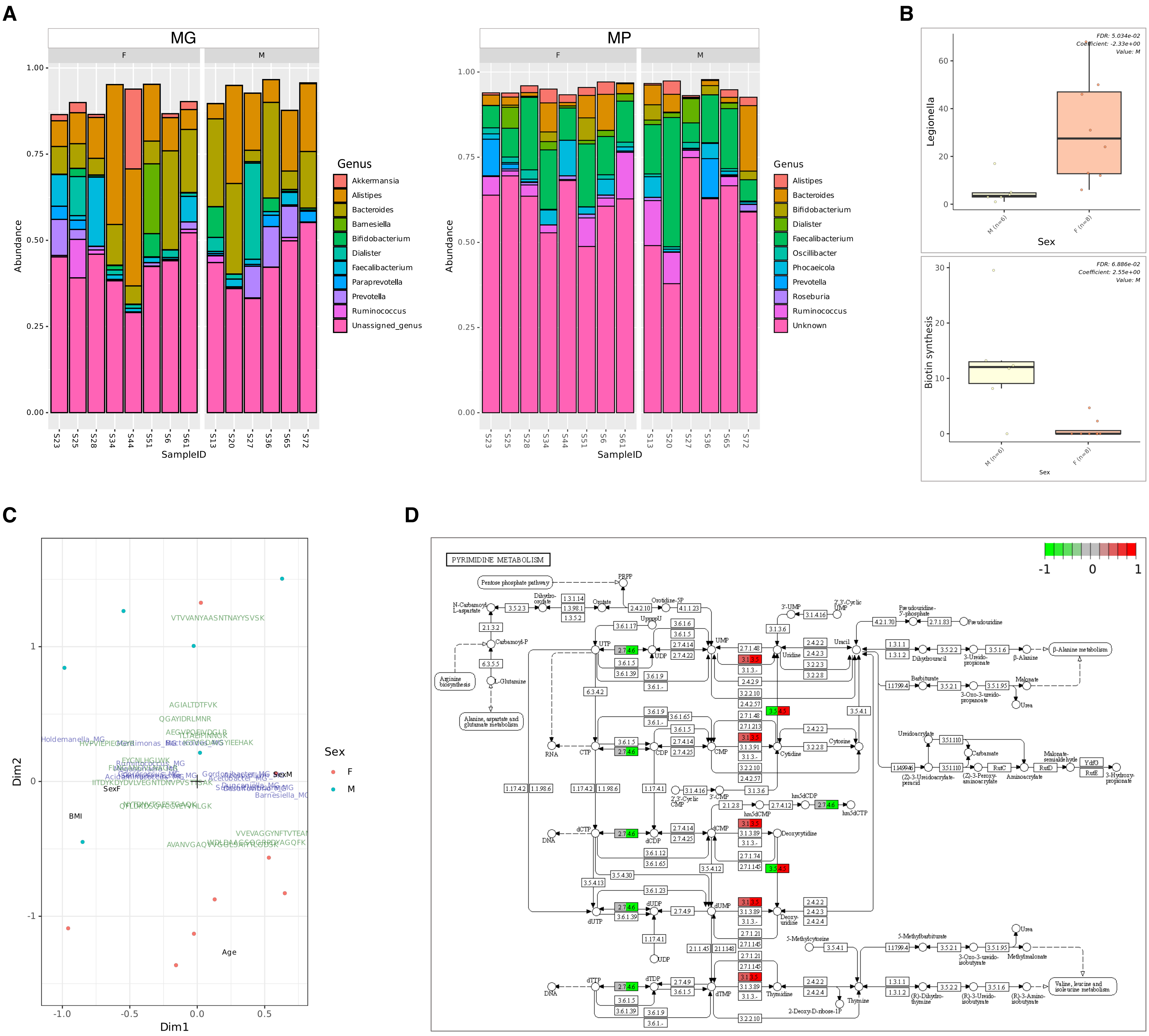

Figure 4

Click here to access/download;Figure;Figure_4.pdf

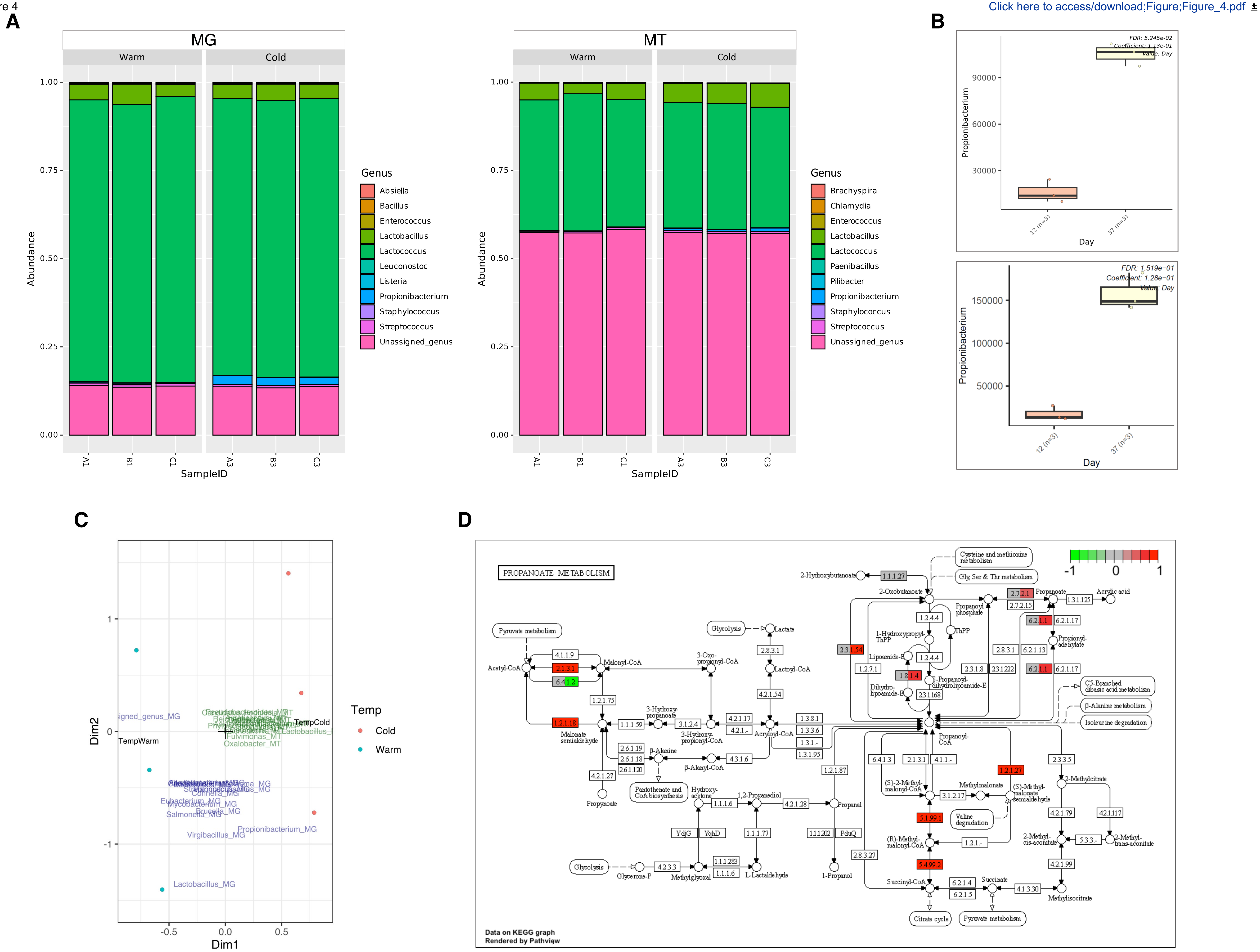

Figure 5

Click here to access/download;Figure;Figure_5.pdf

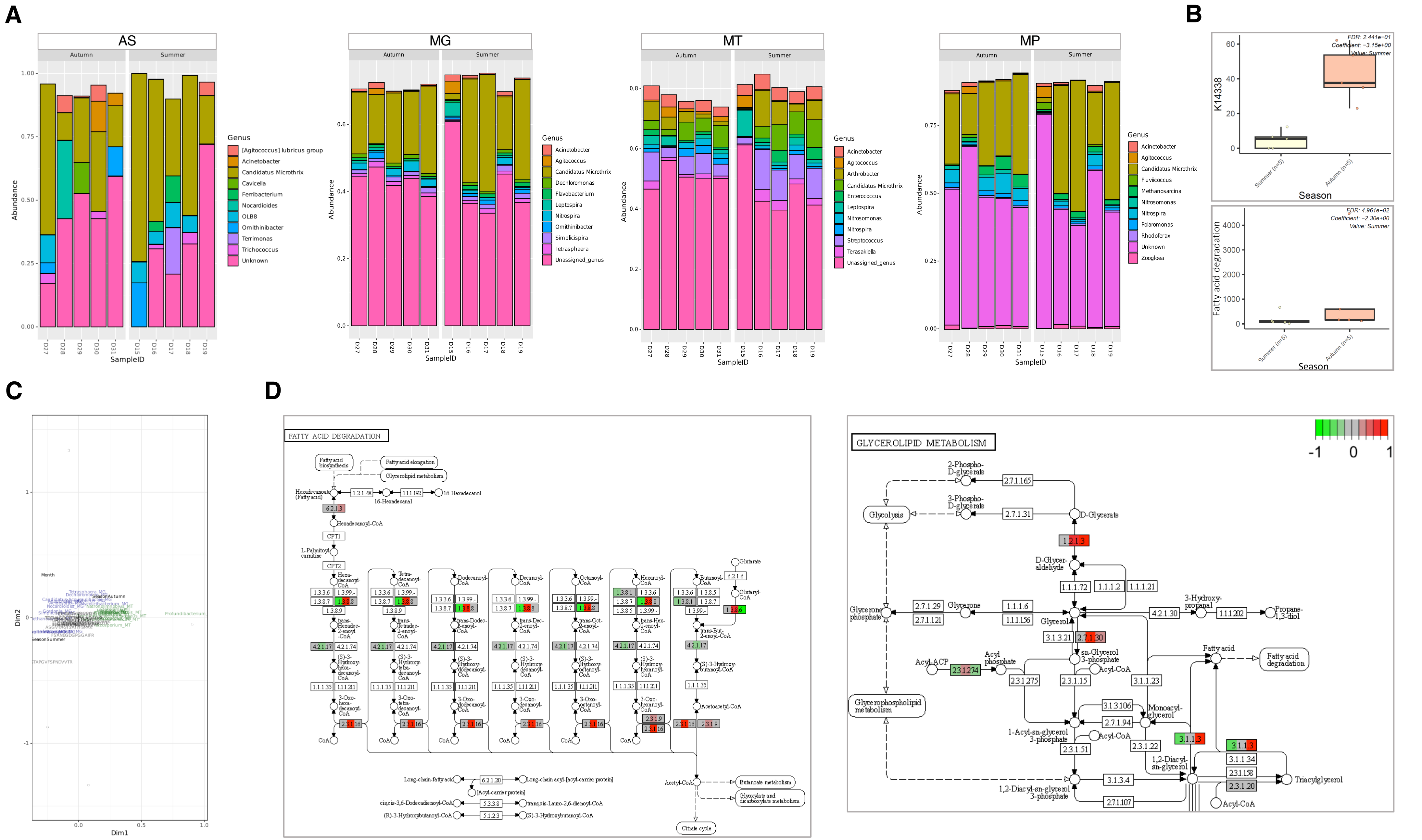

Supplementary Figure S1

Click here to access/download
Supplementary Material
gnomo_vs_gnomo2_v6.png
