# Supplementary figures and images for: gNOMO2: a comprehensive and modular pipeline for integrated multi-omics analyses of microbiomes
Source: Gigascience. 2024 Jul 12;13:giae038. doi: 10.1093/gigascience/giae038 (PMC11240238; doi:10.1093/gigascience/giae038)

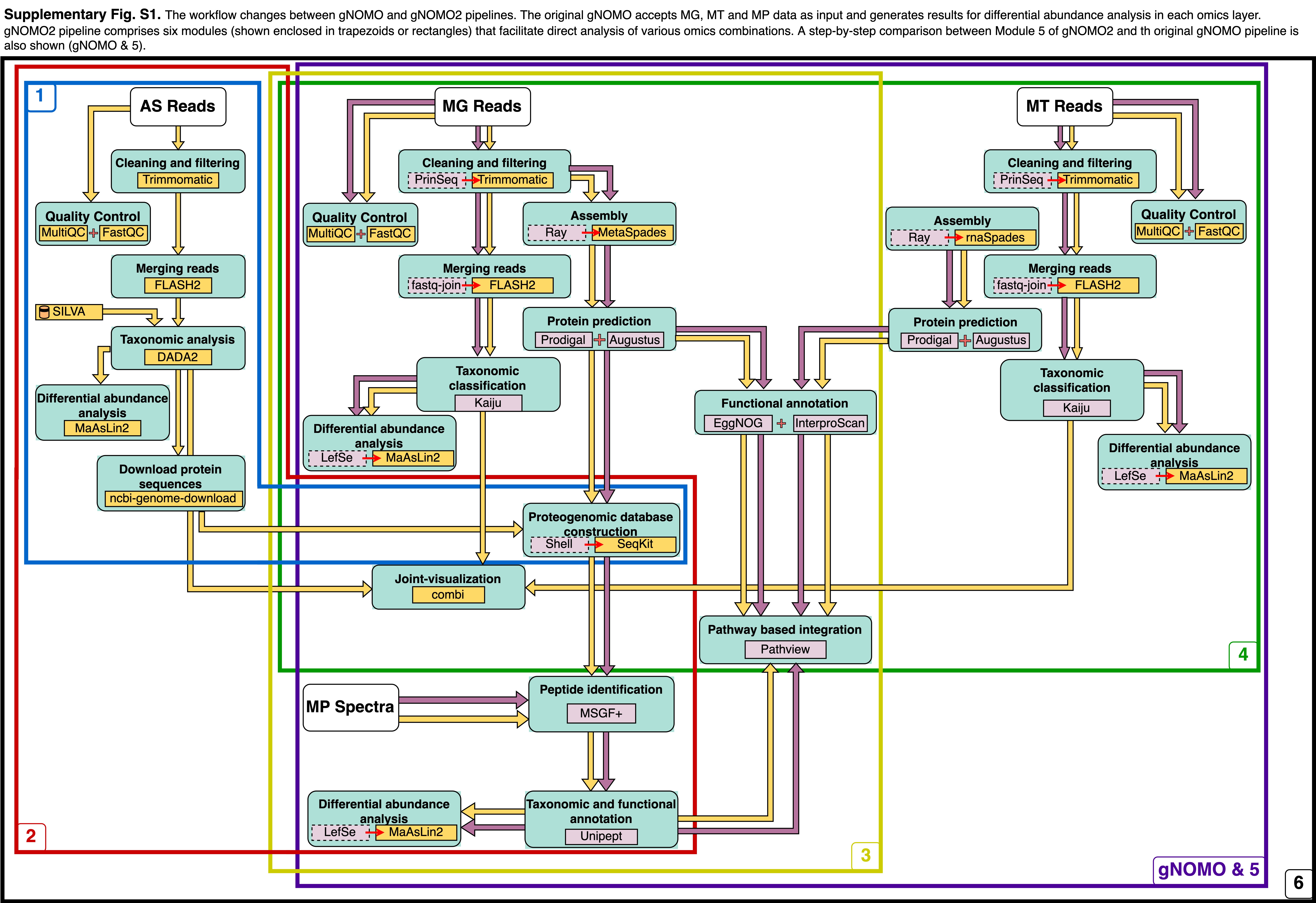

Supplement: giae038_Supplemental_File [file giae038_supplemental_file.jpeg]
