# Supplementary material for: gNOMO2: a comprehensive and modular pipeline for integrated multi-omics analyses of microbiomes
Source: Gigascience. 2024 Jul 12;13:giae038. doi: 10.1093/gigascience/giae038 (PMC11240238; doi:10.1093/gigascience/giae038)
Supplement: giae038_Response_to_Reviewer_Comments_Original_Submission [file giae038_response_to_reviewer_comments_original_submission.pdf]

Author's Response To Reviewer Comments

We would first like to thank the editor and reviewers for their thoughtful and valuable feedback. Please find below our responses to each of comments or questions.

Reviewer #1:
"gNOMO2: a comprehensive and modular pipeline for integrated multi-omics analyses of microbiomes" by Arıkan and Muth presents a multi-omics tools for analysis of prokaryotes. It is an evolution of the first version and offers various separate modules, taking different type of input data. They present different example analysis based on already published data and reproduced the results. The manuscript is very well written (I could not detect a single typo) and it was fun to read! Well done! I have only very few comments and suggestions, see below. However, I had a problem executing the code.

We sincerely thank the Reviewer for taking time to review our manuscript and for valuable comments. Please find below our responses to each of your comments or questions.

################

Key questions to answer:

1) Are the methods appropriate to the aims of the study, are they well described, and are necessary controls included?
Yes
2) Are the conclusions adequately supported by the data shown?
Yes
3) Please indicate the quality of language in the manuscript. Does it require a heavy editing for language and clarity?
Very well written!
4) Are you able to assess all statistics in the manuscript, including the appropriateness of statistical tests used?
No direct statistics given in the manuscript. Maybe the authors could include some example output as .zip file for interested potential users.
We uploaded an example output file for statistical analysis results to GigaDB data repository.

################

Detailed comments to the manuscript:

Line 168: What does "cleaned and redundancies are removed" mean? Are only identical genomes removed? Or are genome part that are identical (I guess this barely exists, except for conserved gene parts as 16S, or similar) removed? Or are only redundant genes removed? How is redundancy defined, 99% identical stretch?
gNOMO2 retains only a single protein sequence from identical protein sequences, effectively removing redundancy among protein sequences that are 100% identical. We revised the sentence as follows:
“All downloaded sequences are merged and cleaned, and a single protein sequence is retained from identical protein sequences to effectively remove redundancy using SeqKit [26].”

Line 399-405: When looking at figure 5A I am wondering how Fluviicoccus and Methanosarcina in the MP faction appear relatively abundant in some samples. Where they de novo assembled in the MG or MT modules?
We identified Fluviicoccus and Methanosarcina in both the MG and MT datasets. However, their relative abundances were comparatively low and thus are not displayed in Figure 5A, which showcases only the top 10 most abundant taxa. However, the taxonomic abundance tables based on MG and MT have been uploaded to the GigaDB data repository with file names “herold_taxa_abundance_mg.txt” and

“herold_taxa_abundance_mt.txt”, respectively.

General comment figures: I know that it is a hack to deal with automatic figure generation and especially the axis labels (as names have very different length). However, I think some figures might be hardly visible in the printed version, especially axes label for panel B are very small. Maybe you can put the critical figures separately in the supplement, e.g. each B panel a one page.
We have uploaded each panel of the figures separately as high-quality figures to GigaDB data repository.

Suggestions: As suggest above, maybe the authors could include some example output (a simple example) as .zip file for interested potential users. This would give an idea of how the output looks like and what to expect besides the plots. But differential abundance tables might be more important than the plots, as the user would generate their own plot for later publications.
We have provided plots, metadata tables, phyloseq objects containing sample data, taxonomy tables, and abundance tables for each dataset analyzed in our manuscript as supplementary data files to GigaDB repository.

################

Github & software:

I also tested the software and followed the instructions in the Github. I successfully executed the "Requirements" and "Config" steps (including create of metadata file and copying of amplicon reads) and tried to execute Modul1. However, the following error occurred (using up-to-date conda and snakemake on Ubuntu linux):

(snakemake) abartho@gmbs17:~/review_papers/GigaScience/gNOMO2$ snakemake -v
6.15.5

(snakemake) abartho@gmbs17:~/review_papers/GigaScience/gNOMO2$ snakemake -s workflow/Snakefile --cores 20
SyntaxError in line 9 of /home/abartho/miniconda3/envs/snakemake/lib/python3.6/site-packages/smart_open/s3.py:
future feature annotations is not defined (s3.py, line 9)
File "/home/abartho/miniconda3/envs/snakemake/lib/python3.6/site-packages/smart_open/__init__.py", line 34, in
File "/home/abartho/miniconda3/envs/snakemake/lib/python3.6/site-packages/smart_open/smart_open_lib.py", line 35, in
File "/home/abartho/miniconda3/envs/snakemake/lib/python3.6/site-packages/smart_open/doctools.py", line 21, in
File "/home/abartho/miniconda3/envs/snakemake/lib/python3.6/site-packages/smart_open/transport.py", line 104, in
File "/home/abartho/miniconda3/envs/snakemake/lib/python3.6/site-packages/smart_open/transport.py", line 49, in register_transport
File "/home/abartho/miniconda3/envs/snakemake/lib/python3.6/importlib/__init__.py", line 126, in import_module

In addition to solving the problem, an example metadata file and some explanation about the output (which I did not see yet) would be good for less experienced users.
This error suggests that the Python version you are using (3.6) does not support future annotations, which were introduced in Python 3.7. Snakemake versions up to 6.0.6 are compatible with Python 3.6. Starting from version 6.1.0, Snakemake requires Python 3.7 or later. Your Snakemake version (6.15.5) is incompatible with Python 3.6 installed on your system. To resolve this, you can upgrade your Python version to 3.7 or later. Additionally, we have updated the gNOMO2 code to require a specific version of Snakemake instead of a minimum version to avoid any other potential compatibility problems.

Reviewer #2:

This paper introduced gNOMO2, a new version of gNOMO, which is a bioinformatic pipeline for multiomic management and analysis of microbiomes. The authors claimed that gNOMO2 incorporates new differential abundance, integration, and visualization tools compared to gNOMO. However, these new features as well as the distinction between gNOMO2 and gNOMO has not been clearly presented in the paper. In addition, the Methods section is written as a pipeline of bioinformatic tools and it is not clear what these tools are used for unless one is familiar with all the bioinformatic tools.
We sincerely thank the Reviewer for taking time to review our manuscript and for providing valuable comments. Please find below our responses to each of your comments or questions.

My major comments are as follows:

1. Given the existing work on gNOMO, it is critical for the authors to distinguish gNOMO2 from gNOMO to show its novelty. In the Methods section, the authors presented the six modules of gNOMO2. Are these all new from gNOMO, or does gNOMO included some of these functions? A clearer presentation of gNOMO2 versus gNOMO is needed.
We have included the following paragraph in the Methods section to elucidate the distinctions between gNOMO and gNOMO2:
“The original gNOMO accepts MG, MT and MP data as input and generates results for differential abundance analysis in each omics layer. It also constructs a protein database using MG and MT data and performs both differential abundance and pathway level integrated analyses (Figure 1A). In contrast, gNOMO2 pipeline comprises six modules that facilitate direct analysis of various omics combinations. Each module includes pre-processing, analysis of each omics dataset, data integration and visualization steps (Figure 1B). We implemented changes to both the analysis workflow and pipeline structure. For workflow adjustments, we updated the quality control, merging, assembly, differential abundance, and visualization steps. In the quality control phase, we switched from using PrinSeq to Trimmomatic for cleaning and trimming reads. For read merging, we replaced fastq-join with FLASH to merge paired-end reads. In the assembly step, we transitioned from Ray to metaSPAdes for de novo assembly of metagenomic sequences, and from Ray to rnaSPAdes for de novo assembly of metatranscriptomic sequences. In the differential abundance analysis step, we replaced LefSe with MaAsLin2. For visualization, we replaced Krona with ggplot2 to analyze taxonomic composition, enabling combined visualization of samples. We also replaced LefSe with MaAsLin2 for visualizing the results of differential abundance analysis. For pathway-level analysis results, we kept Pathview unchanged, but for joint-visualization analysis, we used the combi package to visualize outputs. These workflow changes and comparisons between gNOMO and gNOMO2 are depicted in Supplementary Fig. S1.

Additionally, we introduced changes to facilitate the incorporation of metadata tables into analyses and automated the creation of the configuration file. To enhance and update the structure of the original gNOMO, we implemented six modules in the new gNOMO2 pipeline, allowing for the processing of different omics combinations. The original gNOMO pipeline consisted of only one module (Module 5 in gNOMO2), while gNOMO2 introduced five more modules for specific combinations, along with the ability to accept AS data as input.”

We also added Supplementary Fig. S1 to illustrate the step-by-step differences between the analysis workflow conducted by gNOMO and gNOMO2.

2. The authors did not present the methods in each module very well. For example, the authors wrote in Module 2 that "MaAsLin2 [31] is employed to determine differentially abundant taxa based on both AS and MP data. Furthermore, a joint visualization of MP and AS results is performed using the combi R package [32]. The final outputs include AS and MP based abundance tables, results from differential abundance analysis, and joint visualization analysis results." Without reading the references 31 and 32, it is very hard to understand what this module is really doing.
We have revised the Methods section to provide more detailed explanations of the analyses conducted in each module. The tools, analyses, and outputs are now explained more clearly.

3. The authors used the term "integrated multi-omics analysis" in all six modules of gNOMO2. It is not

clear how this terms really means. It reads like that it is not really integrated analysis, instead, it is more like a module that can handle different types of data separately, such as differential abundance analysis for each type. What other integration has been used except joint visualization? What new integration tools have been incorporated in gNOMO2?
gNOMO2 employs both middle and late integration strategies in its multi-omics microbiome analysis. Pathway and differential abundance analysis-based integrative approaches are considered late integration strategies, where each omics dataset is analyzed separately and then the results are combined. These analyses are utilized in modules 3-6. In contrast, joint-visualization based integration is a middle integration approach that integrates different omics data according to a predefined model, analyzes them together, and generates the outputs. This approach is employed in all modules except Module 1. Additionally, gNOMO2 incorporates integrative analysis of nucleic acid sequencing-based omics methods with metaproteomics data by creating a proteogenomic database, which is utilized in modules 2-6. While the specific integrative analysis methods vary between modules, gNOMO2 ensures that at least one integrative analysis is included in every module.

4. In the differential abundance analysis, does the pipeline consider the features of microbiome data, such as their count, sparsity, and compositional features? Can the modules incorporate covariates in their differential abundance analysis? It is quite useful to have covariates adjusted in a differential abundance analysis?
We have updated gNOMO2 with a new feature in the configuration file that enables users to select the normalization (Choices: "TSS", "CLR", "CSS", "NONE", "TMM") or transformation (Choices: "LOG", "LOGIT", "AST", "NONE") to apply prior to conducting the differential abundance analysis. Additionally, we have introduced a new parameter that allows users to include covariates listed in the metadata table in the differential abundance analysis.

5. In the Analyses section, the authors applied gNOMO2 to re-analyze samples from previously published studies. They found some discrepancy between their results and the ones in the literature. Although some discrepancy is normal, the authors need to explain better what causes the discrepancy and whether it could yield different biological conclusions.
We detected some discrepancies between gNOMO2 results and those reported by Duru et al. (2018) and Herold et al. (2020). To address this, we have added the following paragraphs to the Analyses section to explain better the potential reasons and consequences for these discrepancies:

For Duru et. al (2018):
“Our findings emphasize the critical role of accurately interpreting analysis outcomes based on the structure of the analytical pipeline. Assuming a default approach, particularly during comparison steps, could lead to unsupported conclusions. In meta-omics studies, various approaches can be employed for data analysis. While none of these approaches are inherently wrong, they may not align with the goals set by the research group. When the pipeline's structure is well-defined, no inconsistencies in biological conclusions would be expected. Additionally, we stress the importance of clear language in explaining results in research articles, as failure to do so may mislead readers. In this instance, the discrepancy was primarily due to differences between the approach depending on comparisons at individual MAG level and the gNOMO2 approach, which compares with the whole community.”

For Herold et. al (2020):
“Our findings highlight that read-based and MAG-based taxonomic composition analysis approaches can lead to divergent results and interpretations. Since neither approach is inherently wrong, this disparity underscores the significance and advantage of thoroughly examining meta-omics datasets using various methodologies. Hence, we underscore that employing diverse approaches and perspectives in complex multi-omics datasets may reveal novel insights extending beyond the original hypothesis.”
