# Supplementary material for: gNOMO2: a comprehensive and modular pipeline for integrated multi-omics analyses of microbiomes
Source: Gigascience. 2024 Jul 12;13:giae038. doi: 10.1093/gigascience/giae038 (PMC11240238; doi:10.1093/gigascience/giae038)
Supplement: giae038_Response_to_Reviewer_Comments_Revision_1 [file giae038_response_to_reviewer_comments_revision_1.pdf]

Author's Response To Reviewer Comments

Dear Editor,

Thank you for your feedback. We have incorporated the suggested revisions throughout the manuscript:

1. We have moved the URLs from the main text to the reference list and cited them accordingly.
2. We have added RRID numbers for the tools mentioned in the manuscript.
3. We have included a GigaDB reference for the gNOMO2 supporting data.
4. We have uploaded high-quality figures.

Sincerely,
