# Supplementary material for: gNOMO2: a comprehensive and modular pipeline for integrated multi-omics analyses of microbiomes
Source: Gigascience. 2024 Jul 12;13:giae038. doi: 10.1093/gigascience/giae038 (PMC11240238; doi:10.1093/gigascience/giae038)
Supplement: giae038_Reviewer_1_Report_Original_Submission — Alexander BartholomÃ¤us, PhD -- 3/22/2024 Reviewed [file giae038_reviewer_1_report_original_submission.pdf]

Reviewer Report

Title: gNOMO2: a comprehensive and modular pipeline for integrated multi-omics analyses of microbiomes

Version: Original Submission **Date:** 3/22/2024

Reviewer name: Alexander BartholomÃ¤us, PhD

Reviewer Comments to Author:

Summary:
"gNOMO2: a comprehensive and modular pipeline for integrated multi-omics analyses of microbiomes" by ArÄ±kan and Muth presents a multi-omics tools for analysis of prokaryotes. It is an evolution of the first version and offers various separate modules, taking different type of input data. They present different example analysis based on already published data and reproduced the results. The manuscript is very well written (I could not detect a single typo) and it was fun to read! Well done! I have only very few comments and suggestions, see below. However, I had a problem executing the code.
################
Key questions to answer:
1) Are the methods appropriate to the aims of the study, are they well described, and are necessary controls included?
Yes
2) Are the conclusions adequately supported by the data shown?
Yes
3) Please indicate the quality of language in the manuscript. Does it require a heavy editing for language and clarity?
Very well written!
4) Are you able to assess all statistics in the manuscript, including the appropriateness of statistical tests used?
No direct statistics given in the manuscript. Maybe the authors could include some example output as .zip file for interested potential users.
################
Detailed comments to the manuscript:
Line 168: What does "cleaned and redundancies are removed" mean? Are only identical genomes removed? Or are genome part that are identical (I guess this barely exists, except for conserved gene parts as 16S, or similar) removed? Or are only redundant genes removed? How is redundancy defined, 99% identical stretch?
Line 399-405: When looking at figure 5A I am wondering how Fluviicoccus and Methanosarcina in the MP faction appear relatively abundant in some samples. Where they de novo assembled in the MG or MT modules?
General comment figures: I know that it is a hack to deal with automatic figure generation and especially the axis labels (as names have very different length). However, I think some figures might be hardly

visable in the printed
version, especially axes label for panel B are very small. Maybe you can put the critical figures separately in the supplement, e.g. each B panel a one page.
Suggestions: As suggest above, maybe the authors could include some example output (a simple example) as .zip file for interested potential users. This would give an idea of how the output looks like and what to expect besides the plots. But differential abundance tables might be more important than the plots, as the user would generate their own plot for later publications.
################
Github & software:
I also tested the software and followed the instructions in the Github. I successfully executed the "Requirements" and "Config" steps (including create of metadata file and copying of amplicon reads) and tried to execute Modul1. However, the following error occurred (using up-to-date conda and snakemake on Ubuntu linux):
(snakemake) abartho@gmbs17:~/review_papers/GigaScience/gNOMO2$ snakemake -v
6.15.5
(snakemake) abartho@gmbs17:~/review_papers/GigaScience/gNOMO2$ snakemake -s workflow/Snakefile --cores 20
SyntaxError in line 9 of /home/abartho/miniconda3/envs/snakemake/lib/python3.6/site-packages/smart_open/s3.py:
future feature annotations is not defined (s3.py, line 9)
File "/home/abartho/miniconda3/envs/snakemake/lib/python3.6/site-packages/smart_open/__init__.py", line 34, in <module>
File "/home/abartho/miniconda3/envs/snakemake/lib/python3.6/site-packages/smart_open/smart_open_lib.py", line 35, in <module>
File "/home/abartho/miniconda3/envs/snakemake/lib/python3.6/site-packages/smart_open/doctools.py", line 21, in <module>
File "/home/abartho/miniconda3/envs/snakemake/lib/python3.6/site-packages/smart_open/transport.py", line 104, in <module>
File "/home/abartho/miniconda3/envs/snakemake/lib/python3.6/site-packages/smart_open/transport.py", line 49, in register_transport
File "/home/abartho/miniconda3/envs/snakemake/lib/python3.6/importlib/__init__.py", line 126, in import_module
In addition to solving the problem, an example metadata file and some explanation about the output (which I did not see yet) would be good for less experienced users.

Methods

Are the methods appropriate to the aims of the study, are they well described, and are necessary controls included? Choose an item.

Conclusions

Are the conclusions adequately supported by the data shown? Choose an item.

Reporting Standards

Does the manuscript adhere to the journal's guidelines on minimum standards of reporting? Choose an item.

Choose an item.

Statistics

Are you able to assess all statistics in the manuscript, including the appropriateness of statistical tests used? Choose an item.

Quality of Written English

Please indicate the quality of language in the manuscript: Choose an item.

Declaration of Competing Interests

Please complete a declaration of competing interests, considering the following questions:

- Have you in the past five years received reimbursements, fees, funding, or salary from an organisation that may in any way gain or lose financially from the publication of this manuscript, either now or in the future?
- Do you hold any stocks or shares in an organisation that may in any way gain or lose financially from the publication of this manuscript, either now or in the future?
- Do you hold or are you currently applying for any patents relating to the content of the manuscript?
- Have you received reimbursements, fees, funding, or salary from an organization that holds or has applied for patents relating to the content of the manuscript?
- Do you have any other financial competing interests?
- Do you have any non-financial competing interests in relation to this paper?

If you can answer no to all of the above, write 'I declare that I have no competing interests' below. If your reply is yes to any, please give details below.

I declare that I have no competing interests

I agree to the open peer review policy of the journal. I understand that my name will be included on my report to the authors and, if the manuscript is accepted for publication, my named report including any attachments I upload will be posted on the website along with the authors' responses. I agree for my report to be made available under an Open Access Creative Commons CC-BY license (http://creativecommons.org/licenses/by/4.0/). I understand that any comments which I do not wish to

be included in my named report can be included as confidential comments to the editors, which will not be published.

Choose an item.

To further support our reviewers, we have joined with Publons, where you can gain additional credit to further highlight your hard work (see: https://publons.com/journal/530/gigascience). On publication of this paper, your review will be automatically added to Publons, you can then choose whether or not to claim your Publons credit. I understand this statement.

Yes Choose an item.
