# Supplementary material for: gNOMO2: a comprehensive and modular pipeline for integrated multi-omics analyses of microbiomes
Source: Gigascience. 2024 Jul 12;13:giae038. doi: 10.1093/gigascience/giae038 (PMC11240238; doi:10.1093/gigascience/giae038)
Supplement: giae038_Reviewer_2_Report_Original_Submission — Yuan Jiang -- 3/26/2024 Reviewed [file giae038_reviewer_2_report_original_submission.pdf]

Reviewer Report

Title: gNOMO2: a comprehensive and modular pipeline for integrated multi-omics analyses of microbiomes

Version: Original Submission **Date:** 3/26/2024

Reviewer name: Yuan Jiang

Reviewer Comments to Author:

Referee Report for "gNOMO2: a comprehensive and modular pipeline for integrated multi-omics analyses of microbiomes"
This paper introduced gNOMO2, a new version of gNOMO, which is a bioinformatic pipeline for multiomic management and analysis of microbiomes. The authors claimed that gNOMO2 incorporates new differential abundance, integration, and visualization tools compared to gNOMO. However, these new features as well as the distinction between gNOMO2 and gNOMO has not been clearly presented in the paper. In addition, the Methods section is written as a pipeline of bioinformatic tools and it is not clear what these tools are used for unless one is familiar with all the bioinformatic tools.
My major comments are as follows:
1. Given the existing work on gNOMO, it is critical for the authors to distinguish gNOMO2 from gNOMO to show its novelty. In the Methods section, the authors presented the six modules of gNOMO2. Are these all new from gNOMO, or does gNOMO included some of these functions? A clearer presentation of gNOMO2 versus gNOMO is needed.
2. The authors did not present the methods in each module very well. For example, the authors wrote in Module 2 that "MaAsLin2 [31] is employed to determine differentially abundant taxa based on both AS and MP data. Furthermore, a joint visualization of MP and AS results is performed using the combi R package [32]. The final outputs include AS and MP based abundance tables, results from differential abundance analysis, and joint visualization analysis results." Without reading the references 31 and 32, it is very hard to understand what this module is really doing.
3. The authors used the term "integrated multi-omics analysis" in all six modules of gNOMO2. It is not clear how this terms really means. It reads like that it is not really integrated analysis, instead, it is more like a module that can handle different types of data separately, such as differential abundance analysis for each type. What other integration has been used except joint visualization? What new integration tools have been incorporated in gNOMO2?
4. In the differential abundance analysis, does the pipeline consider the features of microbiome data, such as their count, sparsity, and compositional features? Can the modules incorporate covariates in their differential abundance analysis? It is quite useful to have covariates adjusted in a differential abundance analysis?
5. In the Analyses section, the authors applied gNOMO2 to re-analyze samples from previously published studies. They found some discrepancy between their results and the ones in the literature. Although some discrepancy is normal, the authors need to explain better what causes the discrepancy

and whether it could yield different biological conclusions.

Methods

Are the methods appropriate to the aims of the study, are they well described, and are necessary controls included? Choose an item.

Conclusions

Are the conclusions adequately supported by the data shown? Choose an item.

Reporting Standards

Does the manuscript adhere to the journal's guidelines on minimum standards of reporting? Choose an item.

Choose an item.

Statistics

Are you able to assess all statistics in the manuscript, including the appropriateness of statistical tests used? Choose an item.

Quality of Written English

Please indicate the quality of language in the manuscript: Choose an item.

Declaration of Competing Interests

Please complete a declaration of competing interests, considering the following questions:

- Have you in the past five years received reimbursements, fees, funding, or salary from an organisation that may in any way gain or lose financially from the publication of this manuscript, either now or in the future?
- Do you hold any stocks or shares in an organisation that may in any way gain or lose financially from the publication of this manuscript, either now or in the future?
- Do you hold or are you currently applying for any patents relating to the content of the manuscript?
- Have you received reimbursements, fees, funding, or salary from an organization that holds or has applied for patents relating to the content of the manuscript?
- Do you have any other financial competing interests?
- Do you have any non-financial competing interests in relation to this paper?

If you can answer no to all of the above, write 'I declare that I have no competing interests' below. If your reply is yes to any, please give details below.

I declare that I have no competing interests

I agree to the open peer review policy of the journal. I understand that my name will be included on my report to the authors and, if the manuscript is accepted for publication, my named report including any attachments I upload will be posted on the website along with the authors' responses. I agree for my report to be made available under an Open Access Creative Commons CC-BY license (http://creativecommons.org/licenses/by/4.0/). I understand that any comments which I do not wish to be included in my named report can be included as confidential comments to the editors, which will not be published.

Choose an item.

To further support our reviewers, we have joined with Publons, where you can gain additional credit to further highlight your hard work (see: https://publons.com/journal/530/gigascience). On publication of this paper, your review will be automatically added to Publons, you can then choose whether or not to claim your Publons credit. I understand this statement.

Yes Choose an item.
